# Roadmap on plasticity and epigenetics in cancer

**DOI:** 10.1088/1478-3975/ac4ee2

**Published:** 2022-04-18

**Authors:** Jasmine Foo, David Basanta, Russell C Rockne, Carly Strelez, Curran Shah, Kimya Ghaffarian, Shannon M Mumenthaler, Kelly Mitchell, Justin D Lathia, David Frankhouser, Sergio Branciamore, Ya-Huei Kuo, Guido Marcucci, Robert Vander Velde, Andriy Marusyk, Sui Huang, Kishore Hari, Mohit Kumar Jolly, Haralampos Hatzikirou, Kamrine E Poels, Mary E Spilker, Blerta Shtylla, Mark Robertson-Tessi, Alexander R A Anderson

**Affiliations:** 1 School of Mathematics, University of Minnesota, Twin Cities, MN 55455, United States of America; 2 Integrated Mathematical Oncology Department, Moffitt Cancer Center and Research Institute, Tampa, FL 33612, United States of America; 3 Department of Computational and Quantitative Medicine, Division of Mathematical Oncology, City of Hope National Medical Center, Beckman Research Institute, Duarte, CA 91010, United States of America; 4 Lawrence J. Ellison Institute for Transformative Medicine, Los Angeles, CA 90064, United States of America; 5 Division of Medical Oncology, Norris Comprehensive Cancer Center, Keck School of Medicine, University of Southern California, Los Angeles, CA 90033, United States of America; 6 Department of Biomedical Engineering, Viterbi School of Engineering, University of Southern California, Los Angeles, CA 90089, United States of America; 7 Department of Cardiovascular & Metabolic Sciences, Lerner Research Institute, Cleveland Clinic, Cleveland, OH 44195, United States of America; 8 Case Comprehensive Cancer Center, Cleveland, OH 44106, United States of America; 9 Rose Ella Burkhardt Brain Tumor & Neuro-Oncology Center, Cleveland Clinic, Cleveland, OH 44195, United States of America; 10 Department of Population Sciences, City of Hope National Medical Center, Beckman Research Institute, Duarte, CA 91010, United States of America; 11 Department of Hematologic Malignancies Translational Science, City of Hope National Medical Center, Beckman Research Institute, Duarte, CA 91010, United States of America; 12 Department of Cancer Physiology, Moffitt Cancer Center, Tampa, FL 33612, United States of America; 13 Department of Molecular Biology, University of South Florida Health, Tampa, FL 33612, United States of America; 14 Institute for Systems Biology, Seattle, WA 98109, United States of America; 15 Centre for BioSystems Science and Engineering, Indian Institute of Science, 560012 Bangalore, India; 16 Mathematics Department, Khalifa University, PO Box 127788, Abu Dhabi, United Arab Emirates; 17 Centre for Information Services and High Performance Computing, TU Dresden, 01062, Dresden, Germany; 18 Early Clinical Development, Pfizer Worldwide Research and Development and Medical, United States of America; 19 Medicine Design, Pfizer Worldwide Research and Development and Medical, United States of America

**Keywords:** plasticity, cancer, epigenetics

## Abstract

The role of plasticity and epigenetics in shaping cancer evolution and response to therapy has taken center stage with recent technological advances including single cell sequencing. This roadmap article is focused on state-of-the-art mathematical and experimental approaches to interrogate plasticity in cancer, and addresses the following themes and questions: is there a formal overarching framework that encompasses both non-genetic plasticity and mutation-driven somatic evolution? How do we measure and model the role of the microenvironment in influencing/controlling non-genetic plasticity? How can we experimentally study non-genetic plasticity? Which mathematical techniques are required or best suited? What are the clinical and practical applications and implications of these concepts?

## 1. Introduction to the roadmap on plasticity and epigenetics in cancer

FooJasmine^1^BasantaDavid^2^RockneRussell C^3^
1Department of Mathematics, University of Minnesota, United States of America

2Integrated Mathematical Oncology, Moffitt Cancer Center, United States of America

3Department of Computational and Quantitative Medicine, City of Hope, United States of America


Genetic mutations play a key role in cancer progression and the evolution of treatment resistance. But while these mutations provide the substrate for processes driving somatic evolution, evolution also depends on selection at the phenotypic level, which is driven by epigenetic and microenvironmental factors enabling plasticity in tumour cell populations. Exploring the genetic mechanisms in cancer has driven much of the basic research in the last few decades but new techniques such as single cell sequencing and mathematical modelling have allowed scientists to study the role of plasticity and epigenetics in shaping cancer evolution and response to therapy.

In this roadmap we focus on some of these state-of-the-art mathematical and experimental approaches to interrogate plasticity in cancer. Here we summarize the specific contributions from experts and identify current challenges to understanding the mechanisms and role of phenotypic plasticity in cancer progression as well as discussing ways of translating these ideas into therapeutic opportunities.

### 1.1. Experimental challenges and opportunities

One of the key challenges in the study of plasticity lies in **characterizing and defining phenotypic states**. For example, defining cell states with cell surface markers or antibodies with flow cytometry can give us a very different perspective than using more high-dimensional technology such as single cell genomic sequencing. Even after a phenotypic state is defined, it remains difficult to experimentally isolate cells in specific states to study subpopulations and heterogeneity within a given sample. Another layer of complexity is the possibility of a continuum—rather than discrete set-of states. Cells that are in a state of flux, or transition, between phenotypic states are particularly difficult to identify *in vivo*, where states may exist simultaneously or dynamically change due to environmental factors. Critical experimental challenges also include developing approaches to observe the time scale and frequency of state transitions, which are needed to guide experimental designs.

In addition to defining and observing phenotypic states and transitions, understanding the **mechanisms** driving these behaviours, which may change in space and in time, poses another significant challenge. Inter- and intra-cellular signalling, for instance, is a dynamic, feedback-driven process that depends on the cell microenvironment. There is currently a limited ability to measure or quantitatively interrogate potential mechanisms since many model systems either lack or utilize a vastly simplified representation of the cell microenvironment.

To complicate matters even more, cancer **therapies** which directly or indirectly modify epigenetic states may impact transition rates or induce novel, previously uncharacterized states. These challenges constitute opportunities to develop new experimental model systems and methods of interrogation. This is an active area of research and is ripe for innovation, as discussed in sections by Strelez *et al* and Mitchell and Lathia.

### 1.2. Mathematical challenges and opportunities

In contrast to experimental challenges, which require technological advances and cleverly designed experiments, mathematical models are limited by our understanding of biology and the resolution and quality of the experimental data. The development of new mathematical models and theories can be used, in conjunction with experimental and clinical data, to aid in testing hypotheses about characterization of states, heritability and transience, dynamics of populations, directionality preferences, and environmental effects. However, this requires analyses of evolutionary models which reflect known and hypothesized mechanisms of phenotypic switching, to produce model predictions to compare with experimentally observable measurements.

A pressing practical challenge for mathematical modelling in cancer plasticity includes the need for **statistical techniques** to estimate transition rates and characteristics of individual states from bulk data and single time point snapshots of the dynamic system. As shown by Frankhouser *et al*, this is important for estimating parameters and rate constants in order to quantify and characterize the behaviours of individual states, as well as determine critical time scales of transition dynamics.

At a conceptual level, challenges remain in how to **identify and model genetic and non-genetic evolution in cancer**. This distinction is critical to correctly modelling the mechanism(s) of epigenetic states and plasticity. Genetic and non-genetic evolutionary dynamics may differ substantially and have important implications for therapy design. As suggested by Marusyk *et al* and Huang, integrated modelling frameworks reflecting both genetic and non-genetic evolutionary dynamics are crucial for understanding complex tumour behaviours and response to therapy.

### 1.3. Translating to clinical applications

Ultimately, most studies of epigenetic phenotypes and cellular plasticity in cancer are motivated by the goal of improving the application of existing therapies as well as the development of new ones. The two major contributors to mortality in cancer are metastases and therapy resistance, both of which are mediated by plasticity. Hari and Jolly describe how the integration of clinical and experimental data with machine learning and mathematical multiscale modelling is the key to design new rational therapeutic strategies. From a data-centric point of view, Hatzikirou shows how mathematical models can integrate machine learning-based tools with evolutionary theories of cancer evolution to provide opportunities for biological insight and innovation in therapeutic design.

The pharmaceutical industry, tasked with translating these novel scientific advances into practice, has begun to recognize the role of plasticity and epigenetic states in cancer treatment. In particular, industrial research groups more frequently now employ both experimental and mathematical models to rationally design treatment strategies that prevent epigenetically driven resistance or use therapies targeting epigenetic processes. At a practical level in the clinic, there is a pressing need to connect experiments and modelling to understand clinical responses to epigenetic or cell state modifying treatments such as immunotherapies.

As suggested by Poels *et al*, novel treatment strategies are needed to combat drug-resistance driven by tumour plasticity, and mathematical models can be leveraged to inform the design of such strategies. These efforts will require improved monitoring of dynamic tumour responses *in vivo*, which may be enabled by promising new technologies such as detection and analysis of cell-free DNA and circulating tumour cells.

Plasticity not only provides therapeutic challenges but also shows us novel therapeutic targets that have, by and large, been ignored and that could be exploited to improve outcomes. For example, Robertson-Tessi *et al* consider cellular plasticity in the context of bet-hedging strategies employed in tumour cell populations that drive escape from therapy. They suggest that the mechanisms of phenotypic memory in bet-hedging may be targeted to increase the efficacy of primary therapies.

### 1.4. Concluding remarks

Together, the perspectives from leaders in the field featured in this roadmap present a nuanced view of plasticity, highlighting the new experimental techniques to capture epigenetic mechanisms that drive plasticity at the single cell level as well as the mathematical models and methods that enable integration of data with theory. Together, advances in experimental methods and mathematical modelling and analysis help us better understand the therapeutic implications of cell plasticity in cancer.

### Acknowledgments

The authors would like to acknowledge the University of Minnesota and the Institute for Mathematics and its Applications (IMA) for support.

## 2. Diversity of cancer-associated fibroblasts and their influence on cancer progression

StrelezCarly^1^,^2^ShahCurran^1^,^3^GhaffarianKimya^1^MumenthalerShannon M^1^,^2^,^3^
1Lawrence J. Ellison Institute for Transformative Medicine, Los Angeles, CA 90064, United States of America

2Division of Medical Oncology, Norris Comprehensive Cancer Center, Keck School of Medicine, University of Southern California, Los Angeles, CA 90033, United States of America

3Department of Biomedical Engineering, Viterbi School of Engineering, University of Southern California, Los Angeles, CA 90089, United States of America


### 2.1. Status

Cancer-associated fibroblasts (CAFs), the dominant stromal cell type within the tumor microenvironment (TME), have been linked to several tumor promoting mechanisms across cancer types, including increased tumor cell proliferation and invasion, and protection against drug-induced apoptosis [1]. Research surrounding CAFs has historically focused on identifying markers that uniquely define this cell population. Several studies have attempted to target CAF-specific markers in *in vivo* models and clinical trials, yet these attempts have been unsuccessful [1]. Of late, CAF studies have evolved to include sophisticated subpopulation analyses, revealing significant intra- and inter-tumoral CAF heterogeneity. The presence of CAF diversity may explain why the identification of CAF-specific targets has been challenging and unsuccessful in clinical trials. Increasingly, research in the field suggests that certain CAF populations are indicative of poor patient prognosis, confer drug resistance, and increase tumor invasion and metastasis, while other CAF populations are capable of restraining tumor growth, stimulating a pro-inflammatory TME, and predicting response to immunotherapy [2]. While CAF heterogeneity is acknowledged, there remains limited insight into the functional implications of this heterogeneity on cancer progression.

### 2.2. Current and future challenges

Important work is being done to identify CAF subpopulations, yet there is a critical need to connect these findings to an impact on tumor cell behavior. Moreover, it is now appreciated that CAFs are highly plastic and respond to diverse cues from cancer cells and the TME [2]. Rather than existing in a terminally differentiated state, CAFs can adapt to surrounding factors and interconvert between states, thus influencing tumor cells in a diverse manner. An example of CAF plasticity was recently highlighted in a pancreatic ductal adenocarcinoma (PDAC) study, where the authors identified spatially and functionally distinct CAF subpopulations, termed inflammatory CAFs (iCAFs) and myofibroblasts (myCAFs). They concluded that myCAFs reside closer to the tumor foci, while iCAFs are further away, and an intermediate subpopulation that expresses both myCAF and iCAF markers was also detected. Further work by this group demonstrated that the cell state transition between myCAFs and iCAFs was dependent on TGF-β and IL-1/JAK/STAT signaling from the tumor cells [3]. A deeper understanding of CAF plasticity in the context of tumorigenesis remains an important research challenge with significant clinical implications.

**Figure 1.**
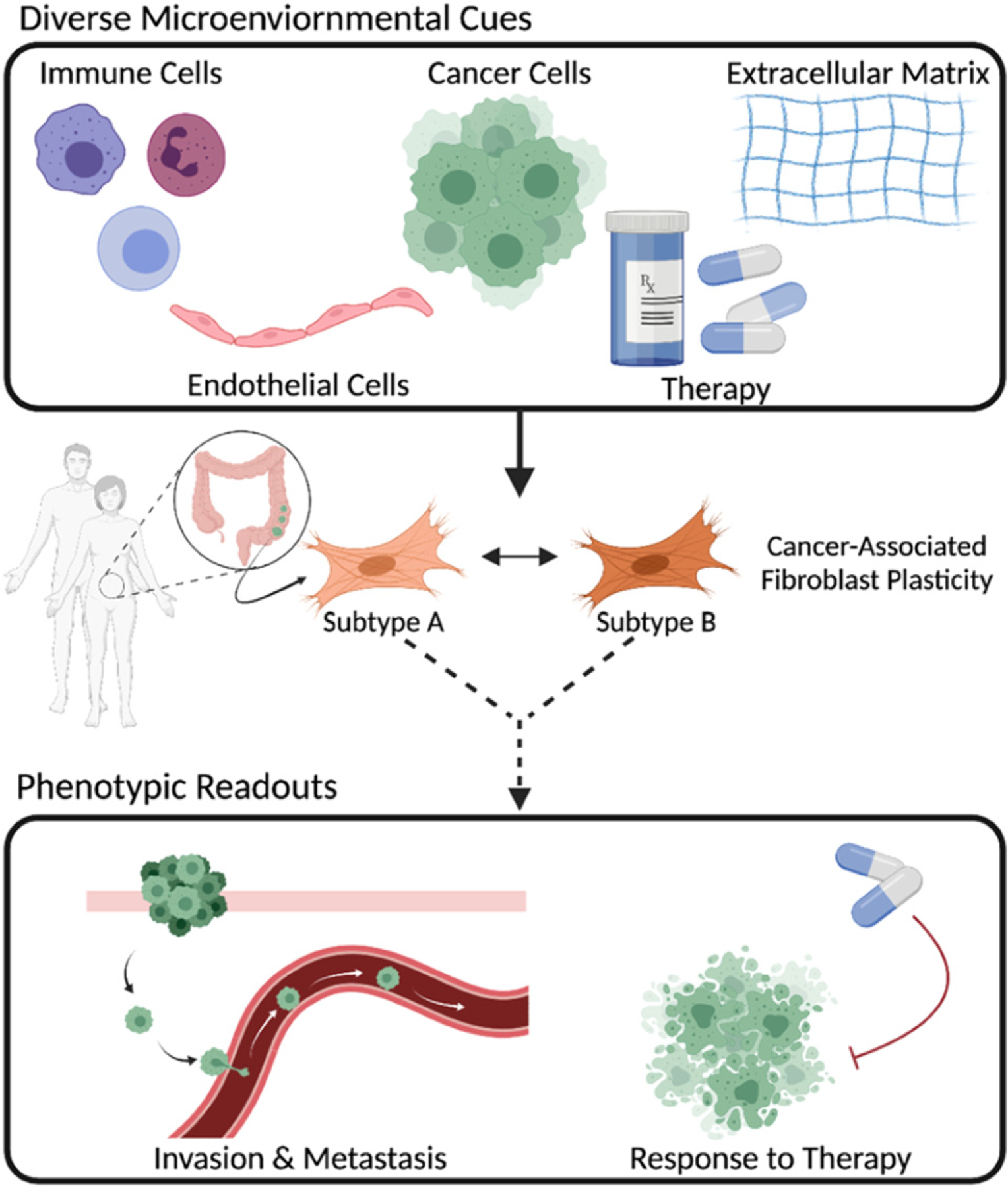
CAFs are comprised of multiple subpopulations that can interconvert based on the cues from the TME. CAF subtypes differentially influence various aspects of cancer progression.

To tackle this challenge, we must address fundamental questions such as: what cues from genetically diverse cancer cells or the TME are responsible for CAFs switching cell states? To what extent does CAF heterogeneity depend on intrinsic mechanisms (e.g., cell of origin) or stochastic gene expression? How do CAF subpopulations evolve over time? How do CAF phenotypic states influence cancer progression? To respond to these questions, it is imperative to recreate and tune aspects of the TME and measure the resulting changes to CAFs and cancer cells (figure 1). This requires (1) biological model systems in which, at a bare minimum, cancer cells and CAFs can be physically cultured together, (2) the ability to deconvolve CAF and cancer cell behavior, (3) measurements that capture spatial and temporal dynamics, (4) development of mathematical models to understand the dynamics at timescales difficult to directly measure, and (5) validation of findings with human tumor tissue.

### 2.3. Advances in science, technology and mathematics to meet challenges

Recent scientific and technological advancements have improved our ability to define and perturb the functional significance of CAF diversity in cancer biology.

*Biomimetic models*: cell culture advances have led to organ-on-chip and spheroid/organoid models that confer 3D spatial relationships in a tunable human TME context while maintaining a degree of throughput previously limited to 2D cell culture studies [4]. The full complexity of organ-level interactions has yet to be captured in these biomimetic models, but the current technologies are conducive to mimicking physical interactions between CAFs and cancer cells and unraveling how CAF populations change in response to the TME [5].

*Experimental tools*: advances in experimental tools have focused on improving resolution from the cell population level to the single cell level. Technologies such as single-cell RNA seq (sc-RNAseq), DNA barcoding, and spatial-omics methods (e.g., seqFISH+) offer interrogation of individual cell transcriptomes, lineage tracing, and a spatial context for the cells, respectively [6–9]. While these technologies tend to generate massive amounts of data that require significant computational power for analysis and individually may lose spatial or full heterogeneity context, they have the potential to provide unparalleled insight into dynamic CAF cell states in a spatiotemporal manner, especially when coupled together.

*Functional readouts*: functional readouts such as tumor cell viability (particularly in response to drug therapies) and migration/invasion assays offer contextual analysis for how CAFs impact tumor cell behavior. Methodological advances in live cell imaging (e.g., light sheet microscopy, time-lapse microscopy, high-content screening), have transformed our ability to visualize diverse behavioral profiles in a spatiotemporal manner at single cell resolutions [10]. While technical considerations must be weighed, including phototoxicity and resolution limitations, advanced imaging and analysis techniques offer greater insight into the functional significance of individual cell states compared to conventional static, population-level readouts. As biomimetic models and assay technologies improve, tumor cell behavior can be studied in a more physiologically relevant context.

*Mathematical modeling*: mathematical models, which are broadly categorized as discrete, continuum, and hybrid, can provide useful predictions of experimental results and offer information at difficult to observe timescales [11]. In one instance, a mathematical model was used to describe the interactions between cancer cells and fibroblasts using nonlinear differential equations to model state transitions between pro- and anti-tumor fibroblasts and investigated the role of phenotypic switching on cancer progression [12]. While mathematical models are immensely powerful, they are inherently biased by the underlying experimental data and theories/principles used to create them. However, this concern can be alleviated via robust experimental protocols that limit experimental bias and maintain the predictive power of the models.

*Integration and applications*: the advances outlined above offer new avenues to interrogate the complexity of CAF phenotypic states and the resulting impact on tumor cell behaviors. For example, in the context of treatment strategies, two studies combining sc-RNAseq analysis of PDAC CAFs with data from patient tissue and clinical trials revealed that some CAF populations can influence tumor response to immunotherapy [13, 14].

**Figure 2.**
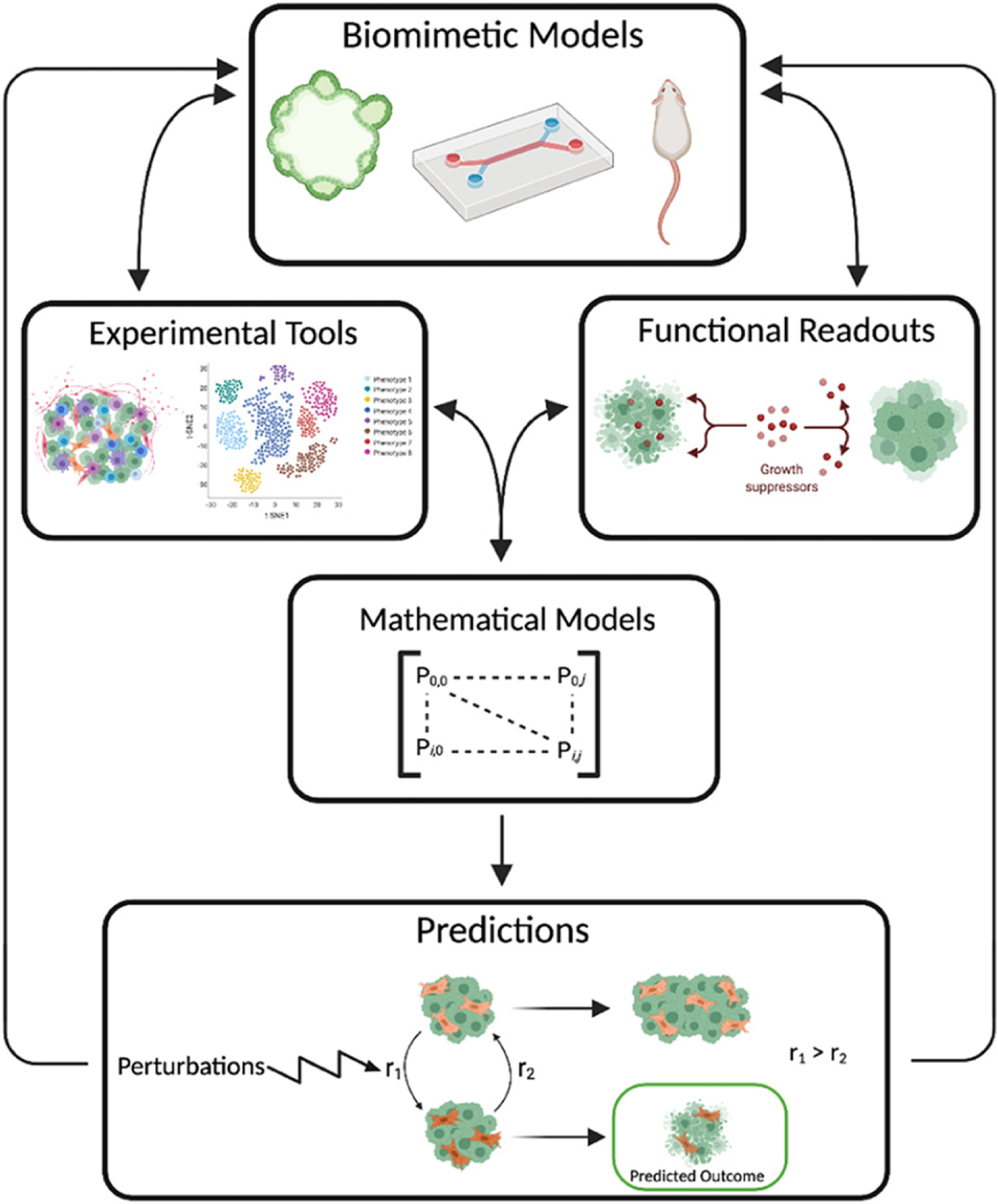
Integration of novel technologies to better understand CAF heterogeneity. Biomimetic models, experimental tools, and functional readouts are used to generate experimental data that can be coupled with mathematical models to make predictions based on model perturbations.

To expand our understanding of CAF plasticity, we suggest a workflow that combines the areas of biomimetic models, experimental tools, functional readouts, and mathematical models (figure 2). For instance, to better study the effect of CAFs on tumor metastasis, a biomimetic organ-on-chip model could be used to accurately recreate the tissue-tissue interface of vessel structures and cancerous tissue while tuning the model to include CAF vs no CAF scenarios [15]. Combining an experimental technique, such as sc-RNAseq of CAFs, with the functional readout of cancer cell intravasation on-chip, measured by live cell imaging, would yield powerful insights into the phenotypic heterogeneity of CAFs and the effects of those phenotypic profiles on invasion. A mathematical model could be developed from the sc-RNAseq and intravasation data and used to generate predictions for how perturbations of the TME influence the composition of CAF subtypes and subsequent tumor cell invasion behaviors. Together, these scientific and technological advances offer unprecedented approaches to studying CAF heterogeneity and, when combined with patient samples and large-scale clinical datasets, can begin to connect the functional relevance of CAF heterogeneity to patient outcome.

### 2.4. Concluding remarks

It is evident that CAFs are important mediators of cell behavior in the TME and although there are significant challenges to studying this cell type, understanding how these cells interact with their surroundings (and systematically targeting these interactions) has the potential to significantly impact patient care.

### Acknowledgments

SMM would like to thank the following funding support: Tissue Engineering Consortium R01 CA241137-01A1 and Cancer Systems Biology Consortium U01 CA232137 at the National Cancer Institute. We would also like to thank J Foo and B Burks for stimulating discussions on modeling cellular plasticity and J Foo, D Basanta, and R Rockne for organizing a thought-provoking workshop on plasticity and epigenetics in cancer evolution.

## 3. Modelling mayhem: interrogating cellular heterogeneity and the cancer stem cell state during tumor progression and in response to therapies

MitchellKelly^1^,^2^LathiaJustin D^1^,^2^,^3^
1Department of Cardiovascular & Metabolic Sciences, Lerner Research Institute, Cleveland Clinic, Cleveland, OH, United States of America

2Case Comprehensive Cancer Center, Cleveland, OH, United States of America

3Rose Ella Burkhardt Brain Tumor & Neuro-Oncology Center, Cleveland Clinic, Cleveland, OH, United States of America


### 3.1. Status

Cellular heterogeneity has long been appreciated as a hallmark of advanced cancers and the significance of these century-old histological observations has been recently confirmed by molecular and functional studies demonstrating distinct cell populations in a given human tumor. First in leukemias, then in breast and glioblastoma (GBM), the demonstration of a cancer stem cell (CSC) population capable of generating heterogeneous tumors from transplantation of a small number of human cells in a xenograft model provided unprecedented opportunities to better understand tumor progression and identify new molecular targets for therapeutic development [16]. While one of the first phenotypes attributed to CSCs was resistance to standard of care approaches, including radiation and chemotherapies, CSCs have been linked to other key oncogenic processes including metastasis, angiogenesis, and immune evasion [17]. More recent studies have demonstrated multiple populations of CSCs in a given tumor with distinct cell cycle states and metabolic dependencies, as well as different underlying gene networks [18], highlighting the circumstance that there is heterogeneity within the CSC compartment. Transcriptional profiling of GBM tumors at single cell resolution has indicated that CSCs likely do not represent distinct populations, but rather a cell state, similar to epithelial and mesenchymal states, and CSCs exhibit plasticity in their capacity to flow in and out of this CSC state. If we maintain that the CSC state is a major contributor to treatment failure, experiments designed to gain mechanistic insight into the cell intrinsic and extrinsic mechanisms controlling transition into and out of the CSC state have the ability to provide much needed information into how tumors develop, progress, and resist therapy.

### 3.2. Current and future challenges

The initial observations of CSCs were based on enrichment via cell surface markers and validation in xenotransplantation and surrogate *in vitro* self-renewal assays. While these approaches have provided a strong foundation for the understanding of CSC properties—namely self-renewal, tumor initiation and recapitulation of tumor heterogeneity—they have been limited in their ability to capture the dynamic and heterogeneous nature of CSCs. For instance, monitoring CSC properties in real time (both *in vitro* and *in vivo*) at high resolution is technically challenging, thereby mechanisms driving cells in and out of the CSC state have been elusive and subject to inference based on static analyses. Some initial progress has been made in this area though the generation of reporter systems such as fluorescent protein promoter reporters of pluripotency transcription factors (NANOG, SOX2, OCT4) shown to be elevated in CSCs [19] or via assessment of the dynamics of CSC division (via symmetric or asymmetric cell division) observed in CSC cultures [20]. These reporter systems have provided some understanding of molecular mechanisms enriched in CSCs and CSC behavior *in vivo*, and lever aging them for high resolution profiling (single cell genomics) or real time tracking in therapeutic contexts *in vivo* could provide deeper understanding of these populations. Specifically, reporter systems could help address the timescale and dynamics of transitions into and out of the CSC state, signals and pressures that induce and reverse these transitions (such as therapeutic pressure and microenvironmental interactions), initial and longer-term clonal diversity of cell types that resist therapy, and the relationship between transcriptional states and reported clonal diversity. Utilization of the abovementioned pluripotent reporter systems may reveal methods to perturb the CSC state through inhibiting plasticity and blocking transitions into the CSC state.

Genetically-engineered mouse models have provided important insight into the mechanisms driving tumor initiation, growth, and progression, and serve as pre-clinical models for therapeutic development efforts, however, the ability to assess the CSC state in real time in these models remains challenging [21]. While immunodeficient models are attractive due to the ability to implant and test therapeutics on primary human tumor cells, given our increased understanding of the importance and coordination of the immune system in GBM, it seems prudent that we also utilize models that recapitulate the immune infiltration and suppressive mechanisms occurring in human tumors [22]. Incorporating these tumor-immune interactions into our experimental modelling of tumor cell states in GBM will be important not only for predicting (immune) therapy clinical outcomes, but also for monitoring CSC dynamics in the context of cell extrinsic pressures. Some exciting progress has been made on this front in brain tumor models (GBM, medulloblastoma) [23, 24], and as with reporter systems described above, there is great potential in using these models for high resolution profiling studies and in-depth *in vivo* assessments.

### 3.3. Advances in science, technology and mathematics to meet challenges

As discussed above, the dynamic nature and clinical relevance of CSCs invoke the need to visualize and track the CSC state in the context of tumor development and in response to therapy, both in conventional mouse models and human-derived models. Below are a summary of advances in CSC systems that have the potential to be leveraged with mathematical modelling to eventually develop, test, and validate integrated therapeutic approaches.

- **Representative models of human cancers and CSC compartment(s):** patient-derived organoid models have led to further understanding of tumor growth dynamics through their increased maintenance of cellular diversity compared to sphere culture, allowing the modeling of CSCs and their non-CSC progeny in parallel. Moreover, the engraftment of tumor-derived organoids into normal human tissue organoids, as has recently been done in GBM models [25–27], provides additional layers of complexity and opportunities to study how tumor cells shape non-tumor niches and vice versa. Organoids provide the opportunity to interrogate the CSC state in an ex vivo system that maintains crucial features of primary tumors such as cellular and microenvironmental heterogeneity, cell-cell interactions, invasiveness and heterogeneous therapeutic response. Such molecular insight can be leveraged for mouse models and next-generation reporter systems.
- **The development of high fidelity CSC reporter systems:** as introduced above, current CSC reporter systems have been built around core pluripotency transcription factors (NANOG, SOX2, OCT4) [19] or cellular processes such as cell division, and provide assessment of the CSC state in real time. Expanding these systems to include lineage tracing elements via genetic approaches to mark individual populations (e.g. confetti [28], MADM [29], ClonTracer [30], macsGESTALT [31]) could be leveraged for powerful dynamic modelling studies. Further, incorporating these reporter systems into organoid and genetic mouse models may vastly improve our understanding of CSC dynamics in the setting of external stimuli.

### 3.4. Concluding remarks

**Figure 3.**
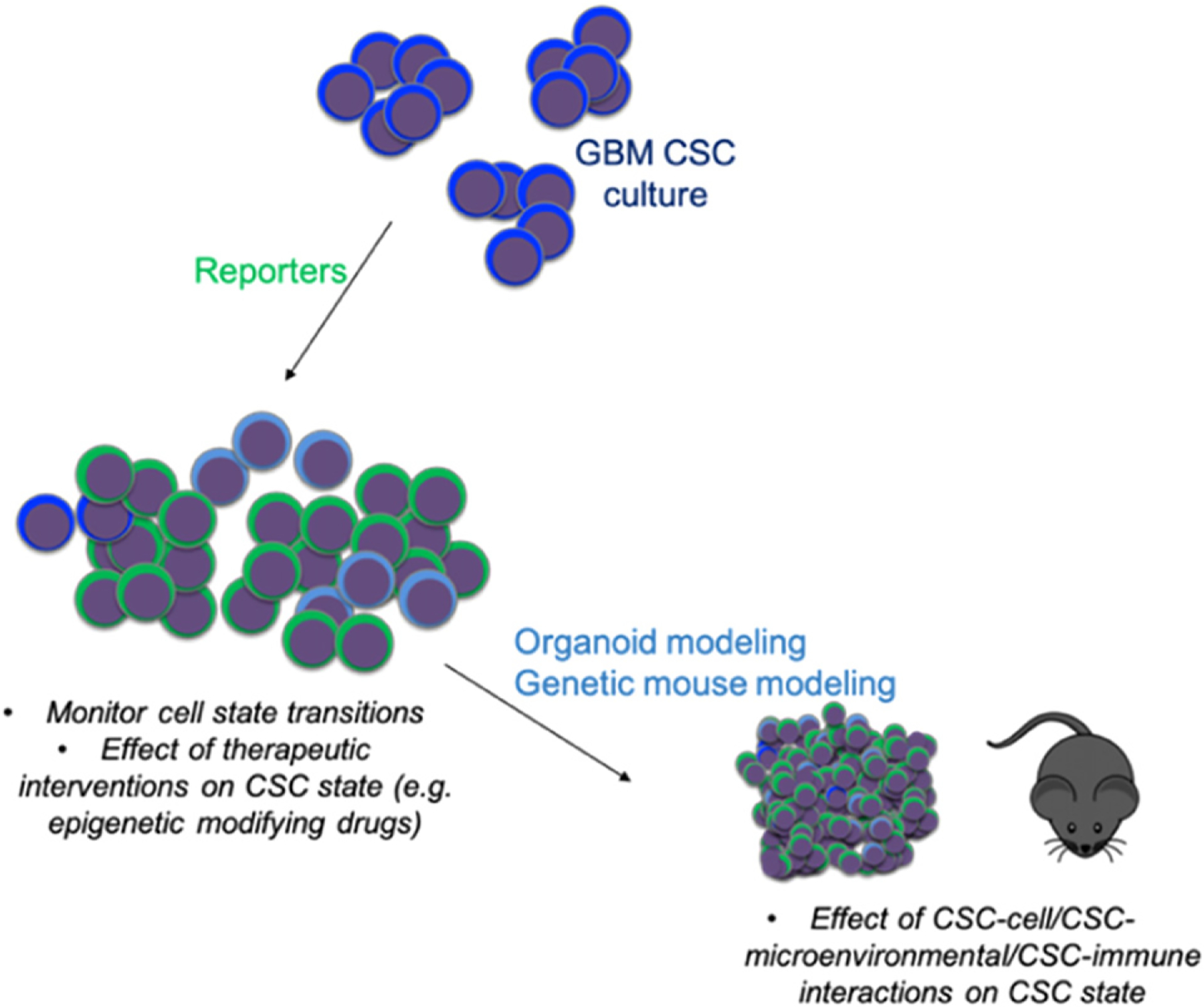
Utilizing reporter systems to track the CSC state in real time in various microenvironmental conditions, therapeutic contexts, organoid models, and *in vivo* could provide valuable insight into development, progression, heterogeneity and therapy resistance in tumors such as GBM.

The cancer biology field continues to experience rapid advances across a variety of areas—ranging from the fundamental understanding of tumor initiation and progression to visualizing tumors at the single cell level—providing great promise for the identification of new treatment strategies. Decades of research support the idea that strategies to compromise the CSC state will likely be therapeutically effective. To facilitate this translational goal, a more nuanced understanding of the CSC state is required and this will require enhanced models, both human-derived and genetically engineered mouse models, as well as the ability to fully appreciate the CSC state in real time *in vitro* and in developing and refractory tumors (*in vivo*). Overall, the future utilization of high-fidelity experimental models in combination with mathematical modelling approaches holds promise for deeper understanding and prediction of the dynamics of CSC state transitions and for the identification of putative vulnerabilities to be exploited for therapeutic benefit (figure 3).

### Acknowledgments

Work in the Lathia laboratory on this topic is currently supported by the Cleveland Clinic, Case Comprehensive Cancer Center, and NIH Grants P01 CA245705, R01 NS109742, and R01 NS117194. Kelly Mitchell is supported by NIH fellowships T32 CA059366, and F32 NS116109.

## 4. Viewing cancer as a system-wide epigenetic state-transition

FrankhouserDavid^1^BranciamoreSergio^2^KuoYa-Huei^3^MarcucciGuido^3^RockneRussell^2^
1Departments of Population Sciences

2Departments of Computational and Quantitative Medicine

3Departments of Hematologic Malignancies Translational Science, Beckman Research Institute, City of Hope, United States of America


### 4.1. Status

**Figure 4.**
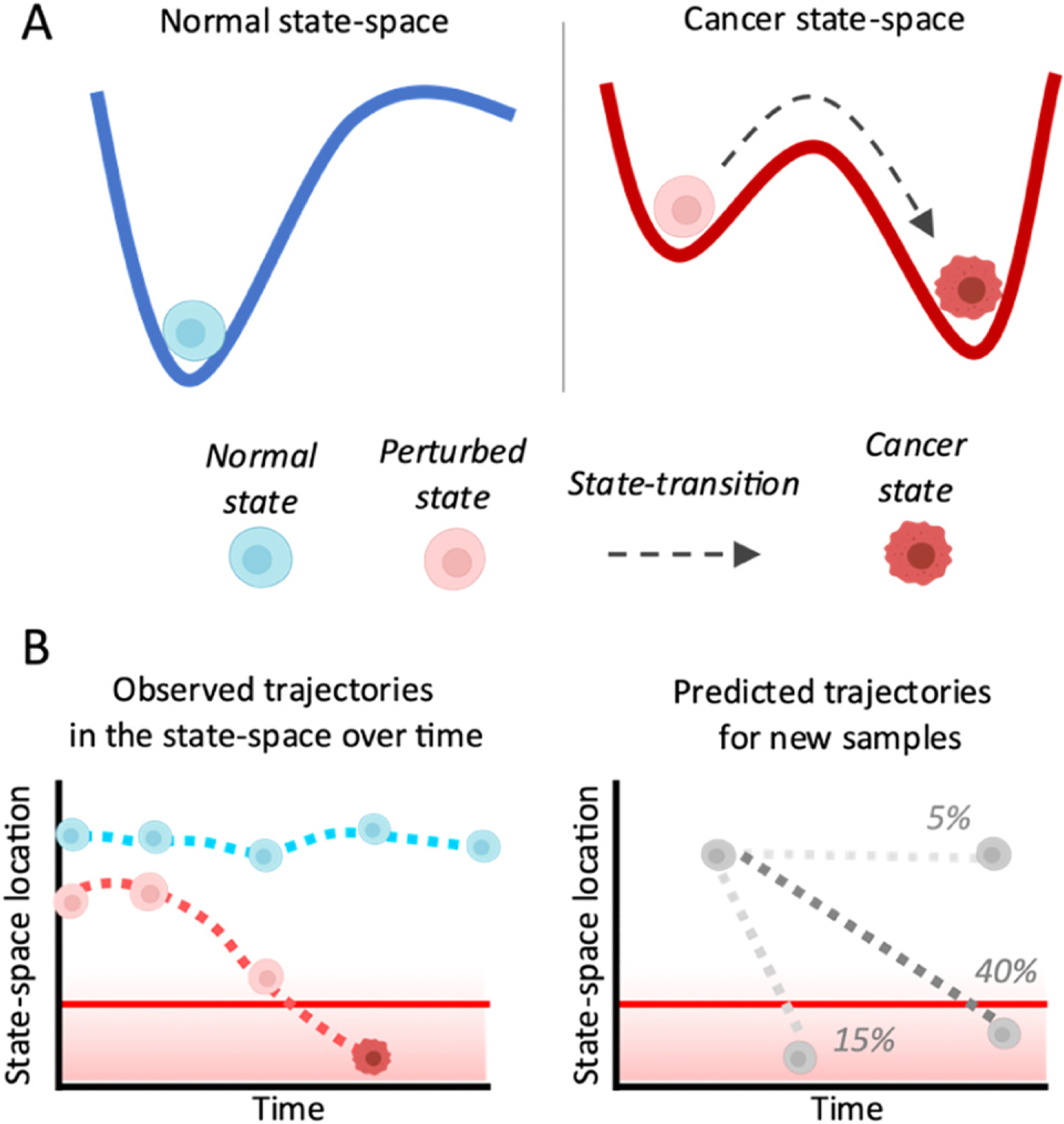
The epigenetic cancer state-space. (A) A phenotypic landscape derived from epigenetic states is shown for normal (left) and cancer (right). The cancer state-space is the normal landscape perturbed by oncogenic events resulting in a lower energy barrier and therefore a higher probability of undergoing a state-transition to the cancer state. In both cases, the evolution of the system is modelled as a particle undergoing Brownian motion in the state-space. (B) (Left) The evolution of the system represented as a trajectory in the state-space over time. The location in the state-space is shown for two samples; one (red samples) that undergoes state-transition to cancer, defined by the red line and one (blue samples) that does not. (Right) Once the state-space is constructed, new samples can be projected into the space to make individual predictions based on the evolution of the probability density function with Fokker-Planck equations corresponding to the equation of motion.

Although cancer is typically viewed as a clonally evolving disease caused by inherited or acquired genetic mutations, epigenetic alterations may also be required for malignant transformation. Epigenetic mechanisms identified in cancer progression include changes in DNA methylation (DNAm), histone modifications, post-transcriptional changes, or altered expression of messenger or micro-RNA (mRNA, miRNA). In this context, an epigenetic landscape represents the set of all possible epigenetic states during transformation from a healthy to diseased state. Consequently, a cancer-specific epigenetic landscape can be viewed as a cancer state-space (figure 4(A)) where cancer evolves through distinct steady states characterized by distinct genetic and epigenetic alterations corresponding to healthy, perturbed, or cancer states. State-space representations have been used to infer cell differentiation and as fitness landscapes in a number of different contexts at the cellular level [32–36], however, these representations often focus on state-transitions of individual cells and not state-transitions of the collection of malignant and non-malignant cells.

The epigenetic state-space approach to studying cancer initiation and progression has many potential research and clinical applications by associating an epigenetic configuration with a specific phenotype. With a state-space constructed empirically with genomic data, biological mechanisms associated with epigenetically driven phenotypic states can be discovered. For example, specific topologies of gene regulatory networks (GRNs) have been shown to produce attractor states in the landscape [37–39].

Clinically, the state-space has diagnostic, predictive, and prognostic potential. By following a patient longitudinally over time to capture sufficient information about the disease state-space, an individual’s disease trajectory can be predicted by solving stochastic equations of motion in the landscape (figure 4(B)). Trajectories in the epigenetic state-space can be used to predict disease progression or response to treatment for an individual patient. Personalised predictions from epigenetic states can add an additional layer of information that clinicians can use to tailor therapeutic decisions to an individual’s predicted course of disease. However, several challenges remain to translate these concepts into the clinic which are outlined in *current and future challenges*.

Acute myeloid leukaemia (AML) is an ideal model system for investigating the dynamics of epigenetic state-transitions of cancer because the disease can be directly assayed through the peripheral blood at sequential time points without invasive procedures. In AML, epigenetic mechanisms of DNAm and miRNA expression play an important role in disease initiation and evolution [40, 41]. Although DNA mutations have been observed years before diagnosis [42, 43] and DNAm has been investigated for its role in AML state-transition [44], miRNA have often been overlooked as an epigenetic mechanism, despite their role in post-transcriptional regulation of onco- and tumour suppressor gene expression. We have shown that mRNA and miRNA expression levels in blood cells—both normal and leukemic cells—can be used to create an AML state-space where it is possible to follow the system undergoing state-transition during AML development, thereby reinforcing the role of epigenetic states for AML pathogenesis and in general, for malignant transformation [32].

### 4.2. Current and future challenges

The most formidable challenge in a state-space approach is constructing and identifying the dimensionality of the state-space. First, the number of stable and unstable states must be observed or hypothesized, which requires multiple sequential observations of the system. Unstable states are less likely to be observed than stable states and therefore their existence must be hypothesized or inferred. Moreover, longitudinally collected samples from individual patients are expensive and difficult to obtain. One approach to address this challenge is to use samples from different individuals at different stages of cancer development to increase the number of observations and create pseudo trajectories; however, genetic variation across individuals makes this approach challenging. An alternative approach is to use a disease model, such as a mouse, where samples can be more easily obtained, from the induction of driver gene mutations and throughout the course of disease development. In addition to the challenge of having a ‘good’ mouse model recapitulating the human disease, we also face the challenge of mapping between mouse and human.

Another challenge is quantitatively mapping the underlying biological mechanisms that produce or alter empirically derived state-spaces. In system-wide state-spaces derived from bulk samples where multiple cell types contribute to the observed epigenetic state, identifying a mechanism is difficult, and mathematically impossible to solve uniquely. To address this challenge, one approach is to compare landscapes constructed with single cell genomic sequencing to the landscape derived from bulk samples. However, single cell experiments present an additional set of challenges; for example, single-cell data can be sparse due to gene dropouts which are false negatives created when certain genes are not observed in all cells. Future studies need to investigate whether similar information regarding the system’s epigenetic state is contained in both single-cell and bulk samples. To this end, we have already shown that changes in gene expression may be detected from bulk samples before either phenotypic changes or leukemic cells can be detected; this supports the concept that cancer initiation may induce system-wide epigenetic state-transitions [32].

### 4.3. Advances in science, technology and mathematics to meet challenges

The most significant advance to enable epigenetic state-space models of cancer is the routine collection of ‘omics’ data at sequential time-points from diagnosis through treatment, which is supported by the precision medicine approach. Although longitudinal collection of genomic data remains mostly in research centres, such data may enable the creation of patient-specific epigenetic state-transition trajectories correlated with clinical features and outcomes. Moreover, the use of standardized sequencing arrays enables quantitative and reproducible state-spaces and trajectories that reduce the variability in genomic data due to sequencing technologies and bioinformatics methods.

In parallel to the collection of genomic data over time, advances in mathematical methods to construct the state-space are equally important. Although not a recent advance, dimensionality reduction methods such as the singular value decomposition (SVD)combined with information-theoretic measures such as mutual information, can be used to quantitatively identify genomic features most relevant to cancer state-transition. Using SVD which produces lower dimensional representations of the data, a state-space can be constructed from one or more principal components; the associated eigenvector(s) describes how each gene contributes to the state-space construction. This enables inference of state-transition dynamics and identification of eigenvectors of gene expression, or ‘eigengenes,’ that contribute to the normal, perturbed, transition, or cancer states [32, 45].

A generalisation of the SVD, the tensor GSVD, provides a method to simultaneously integrate multiple data types, (ex. mRNA, miRNA, DNAm) derived from the same sample to identify novel multi-omic defined epigenetic states [45]. Integrating multiple epigenetic data from bulk samples may provide more accurate predictions of future states and has the potential to reveal multifaceted underlying mechanisms of cancer state-transition. In support of a multi-omic view of epigenetic state-transition, we have shown that an AML state-space can be constructed using bulk miRNA as well as mRNA expression profiles from peripheral blood in a mouse model [35]. Intriguingly, the miRNA-derived state-space is very similar to the mRNA-derived state-space, but not identical. This raises the possibility that mRNA and miRNA contain complementary information about AML induced epigenetic state-transition.

Another advance is the use of data-driven mathematical models to predict the evolution of epigenetic states over time. By identifying critical points in the state-space, an individual’s epigenetic state can be modelled as a particle undergoing Brownian motion in the landscape. Importantly, given the location of a sample in the state-space, the probability of finding the location of the particle at some future time can be predicted with the solution of the Fokker–Planck equation corresponding to the equation of motion in the state-space (figure 4(B)).

Our studies of AML state-transition illustrate how an epigenetic state-space provides an analytical framework to investigate biological processes and predict disease evolution. Since the location in the state-space represents a phenotypic state in cancer development, the state-space can be used to align individuals with different disease states and rates of disease progression. We have leveraged this to investigate biological processes specific to states of cancer development and have discovered nonlinear dynamic patterns of mRNA and miRNA expression that can be used to identify potential therapeutic targets.

### 4.4. Concluding remarks

The conceptualization of cancer as an epigenetic state-transition of the system, beyond the transformation of individual malignant cells and through different states of the disease and treatment response, is a powerful and potentially insightful approach for understanding cancer dynamics that compliments the DNA mutation and clonal evolution centric view. With genomic sequencing becoming more routine in the clinical management of cancer and precision medicine, state-transition models can be a powerful predictive tool to guide the development of therapies that target critical points in epigenetic state-transitions.

### Acknowledgments

This work was supported in part by the NIH under Award Number R01CA178387 (to Y-H Kuo), R01CA205247 (to Y-H Kuo/G Marcucci), U01CA250046 (to R C Rockne, Y-H Kuo, G Marcucci), R01CA248475 (G Marcucci), T32CA221709 (D E Frankhouser), and the Gehr Family Center for Leukemia Research. Research reported in this publication included work performed in the Integrated Genomics Core, Bioinformatics Core, Analytical Cytometry Core, and Animal Resource Center supported by the NCI of the NIH under Award Number P30CA33572. The content is solely the responsibility of the authors and does not necessarily represent the official views of the NIH.

## 5. Integrative understanding of acquired therapy resistance

Vander VeldeRobert^1^,^2^MarusykAndriy^1^
1Moffitt Cancer Center, Department of Cancer Physiology

2USF Health, Department of Molecular Biology, United States of America


### 5.1. Status

A revolution in molecular biology has enabled an explosion of studies leading to an in-depth interrogation of molecular mechanisms that underlie cancer-specific phenotypes (hallmarks) [46]. These studies resulted in the identification of molecular targets, whose suppression induces remarkable clinical responses with minimal toxicities, enabling control of disease over months or even years, such as with inhibitors of mutant EGFR and anaplastic lymphoma kinase (ALK) in lung cancers. Unfortunately, these responses do not translate into cures in advanced metastatic neoplasia. A similar situation is observed in cancers that are treated with more traditional, cytotoxic chemotherapies, where cancers typically relapse despite strong initial responses.

One obvious cause of acquired resistance is that therapies fail to eliminate all tumor cells in advanced metastatic cancers. This failure is the consequence of tumor heterogeneity: genetic and phenotypic differences between individual neoplastic cells in tumor cell populations, as well as differences in TMEs. While most tumor cells can be eliminated by properly selected therapy, some tumor cells can avoid elimination due to cell-intrinsic properties that make them tolerant [47, 48,] or resistant to treatment, or microenvironmental location [49] that limits drug penetration or provides signals that counteract the effects of therapies.

While the key importance of intratumor heterogeneity as a cause of therapy resistance is widely recognised in the field, development of therapy resistance by initially sensitive tumors has another salient cause: the ability of populations of tumor cells to change under therapy-induced selective pressures.

**Figure 5.**
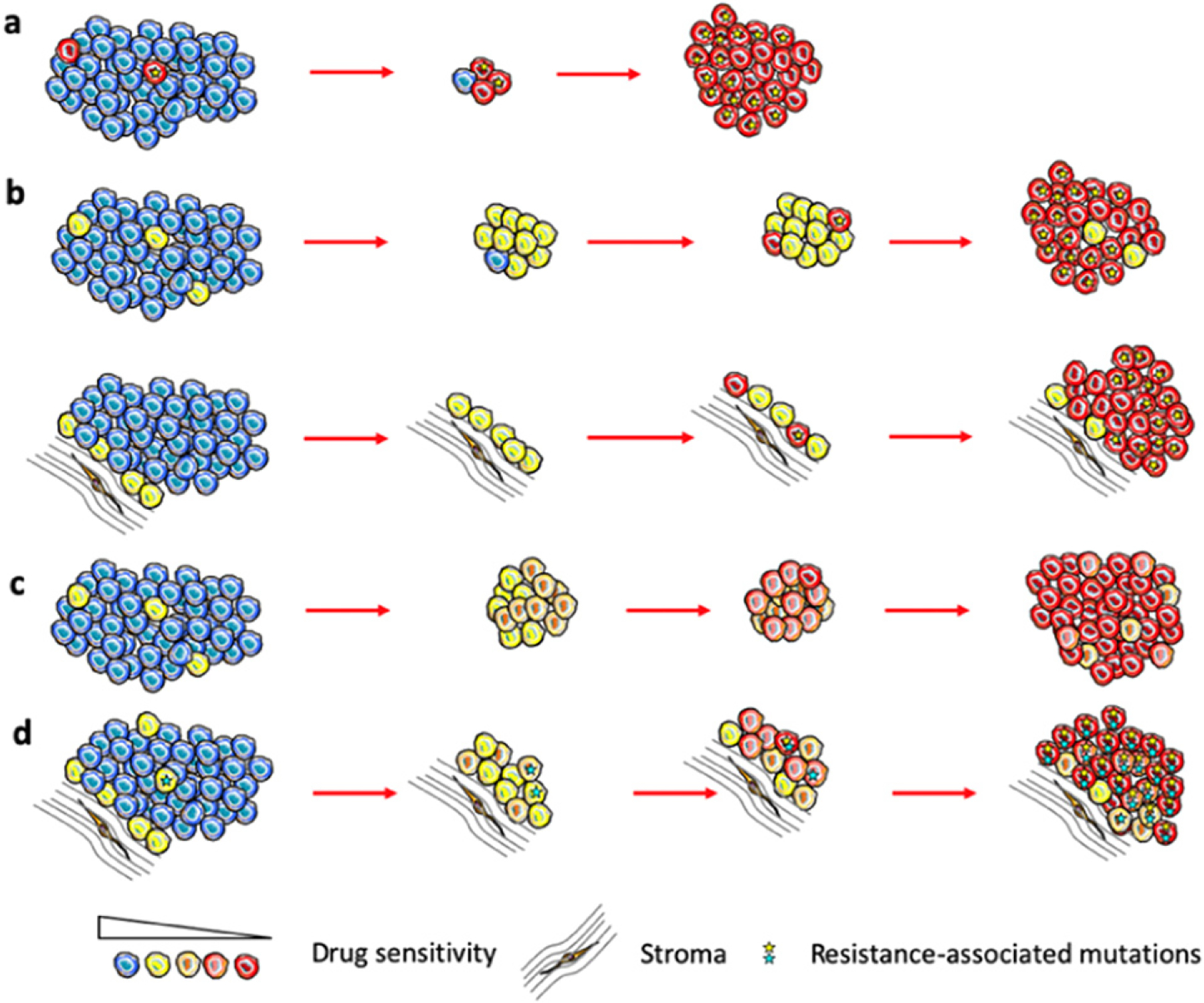
Models of acquired therapy resistance. (A) Pre-existent fully resistant subpopulations expand due to therapy-induced competitive release. (B) Full resistance develops from tolerant cells or cells sheltered from therapy by proximity to protective stromal niches due to stochastic occurrence of resistance-conferring (epi)genetic mutation. (C) Resistance as the result of plasticity-mediated therapy induced phenotypic ‘reprogramming’. (D) Multifactorial, gradual acquisition of resistance resulting from integration of multiple contributing inputs.

In the simplest case, when fully resistant cells are present in a tumor before treatment, therapy causes their competitive release and expansion (figure 5(A)). At some point, this expansion translates into net positive growth of tumors, leading to relapse. On the other hand, undeniable experimental and clinical evidence shows that strong resistance can be acquired by cells that are initially sensitive or weakly resistant (persistent/tolerant) to therapies [50]. Whether resistant cells pre-exist or arise de novo is still a subject of debate. However, it is hard to reconcile pre-existing resistance with remission that lasts for months and years before re-emergence of rapidly growing tumors (most patients on front line therapies in ALK+ and EGFR mutant lung cancers).

### 5.2. Current and future challenges

Arguably, an adequate understanding of how and why resistance develops is prerequisite to developing therapeutic strategies that can achieve substantial improvements in clinical outcomes. While great advances have been made in the understanding of individual resistance mechanisms, our knowledge of how resistance develops from sensitive or weakly resistant cells is very limited. In the case of genetic resistance mechanisms, such as target amplification or point mutations that disrupt binding of the drug, resistance is assumed to be a result of a single stochastic mutational event (figure 5(B)). Emergence of non-genetic resistance mechanisms is less clear. Some evidence points to the possibility of stochastic hardwired epigenetic changes, analogous to genetic mutational events [47]. On the other hand, ample evidence points to the importance of therapy-induced changes, where at least some tumor cells transition to more plastic phenotypic states [36] (frequently referred to as CSCs). While stemness and epithelial to mesenchymal transition (EMT) in carcinomas are commonly considered to be sufficient to fully account for resistance [51], recent studies as well as first principles point to a distinct phenomenon, where phenotypic plasticity enables cells to adjust GRNs, achieving phenotypic states that no longer rely on the activity of the therapeutic target [36, 52, 53] (figure 5(C)).

While epigenetic adaptations to therapy-induced stress clearly fall outside of the conventional Darwinian paradigm of stochastic heritable variability, and have closer parallels to the Lamarckian paradigm [36], the resulting resistant phenotypes can and do act as a substrate for selection forces that act on the population level [52]. Importantly, stochastic (both genetic and epigenetic) and induced changes are not mutually exclusive. Our recent work [54], as well as studies from other groups [53] suggest that therapy resistance represents complex, multi-step adaptations, where resistance reflects a combined contribution of multiple individual resistance mechanisms, including both genetic and epigenetic changes. Moreover, *in vivo*, these changes occur within spatially diverse microenvironmental contexts that can dramatically impact therapeutic sensitivity, phenotypic state transition and evolutionary dynamics (figure 5(D)).

### 5.3. Advances in science, technology and mathematics to meet challenges

We posit that despite undeniable utility, reductionistic studies are not sufficient to provide an adequate understanding of therapy resistance, much like how a catalogue and detailed studies of airplane parts cannot explain how an airplane flies, and increasing resolution of mechanistic detail can only obfuscate the answer. Instead, we need to develop knowledge that integrates the multiple molecular inputs that lead to the development of resistance and understand the spatiotemporal dynamics of this process. Since linear logic is not suitable for this task, the challenge can only be addressed with the help of mathematical modelling tools, even if an appropriate toolset still needs to be fully developed and refined.

Specifically, we will need to understand the process of phenotypic adaptation from a biology perspective as a trajectory across a cell state landscape, incorporating the input of induced and stochastic changes. Similarly, we need to understand evolving resistance as a trajectory on an adaptive landscape in a way that incorporates the impact of mutational and expression level changes that impact cell fitness.

**Figure 6.**
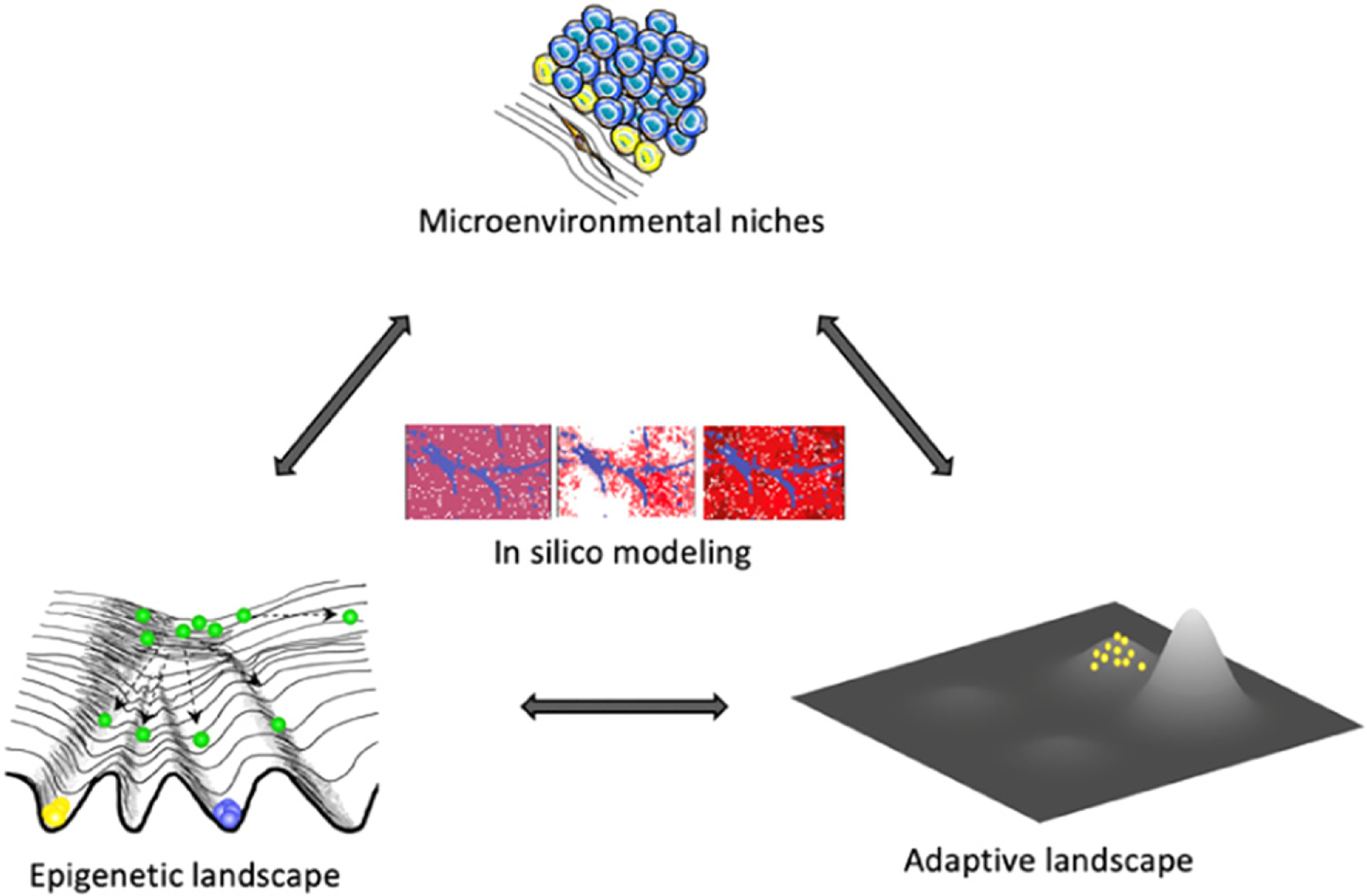
Understanding of acquired resistance requires consideration of epigenetic reprogramming, stochastic genetic and epigenetic changes, converging at the level of inclusive fitness ‘seen’ by selection. Such an integration requires development and use of mathematical modelling tools.

A stiffer, unresolved challenge is to integrate consideration of both cell state and adaptive landscapes, while accounting for the impacts of distinct microenvironmental niches, limited heritability of many of the epigenetic changes, and interactions between evolving subpopulations in space and time (figure 6).

Addressing these challenges is not trivial, as it will require the development and integration of new conceptual frameworks as well as new experimental and modelling pipelines. Moreover, ideal integration of all of the essential determinants of resistance is likely to be unrealistic. Yet, this does not mean that the mission is impossible, as even partial advances in this area could translate into improved ability to optimize therapies towards long term outcomes rather than maximizing short term gains.

### 5.4. Concluding remarks

Adequate understanding of acquired therapy resistance requires acquisition, and modelling assisted integration of knowledge of epigenetic, genetic and microenvironmental determinants of resistance, within evolving neoplastic populations. Achieving meaningful progress in this direction must start with the recognition that the problem of cancer cannot be fully solved within the dominant reductionistic frameworks. At least equal efforts need to be devoted towards the more challenging task of integration. As progress in this area requires development of conceptual frameworks, experimental and modelling tools, as well as fully integrated research teams, it is not realistic to expect a quick fix and immediate translation before adequate understanding is gained. Still, as we build up the knowledge, it will enable progressive development of more effective therapies, using existing and future drugs and treatment modalities.

### Acknowledgments

This work was supported by research Grant from the Moffitt Center for Evolutionary Therapy, and by the Department of Defense LC190085.

## 6. A dynamical systems framework for uniting the Darwinian and Lamarckian schemes of treatment-induced tumor progression and analyzing single-cell omics profiles

HuangSuiInstitute for Systems Biology, Seattle, United States of America

### 6.1. Status

The rapid recurrence of tumors after treatment defies the prevailing Darwinian paradigm of random mutation and selection as driver of progression [36, 55–57]. The recent spate of single-cell resolution gene expression profiles of tumors reveals non-genetic phenotype plasticity of cancer cells that allows regulated cell state transitions into new, inheritable phenotypic states without mutations. This property is at odds with somatic Darwinian evolution of tumor cells. It permits Lamarckian [36] dynamics and calls for cancer biology to embrace principles that govern the process by which the same set of genes collectively produces a variety of discretely distinct stable cell phenotypes, such as the canonical cell types.

Reliance on genetic mutations and a 1:1-*genotype* ↔ *phenotype* correspondence in Darwinian thinking [36] obviates the need for mathematical theory to explain new phenotypes. But how can an invariant genotype (the genome) produce a diversity of phenotypes, i.e. the phenotypic states of cells?

In metazoan the most elementary phenotype is the cell type, commonly defined by a particular configuration (state vector) ***x*** of the expression levels of all the *m* genes of the genome, ***x*** = [*x*_1_, *x*_2,_, ‥, *x*_i_, ‥, *x*_*m*_], which is approximately measurable as the transcriptome. The genome can produce only a particular set of stable configurations ***x*** * (that we observe as phenotype) because genes are not independent but regulate each other via the GRN that is ‘hardwired’ in the genome via the cis and trans regulatory sequences [58]. The GRN is the dynamical system $\dot{x}=F(x)$ that actuates the change of expression, ${\dot{x}}_{i}$ , of gene *i*, and thereby drives the cell state ***x*** towards stable steady-states ***x*** * with ***F*** (***x*** *) = 0, or *attractor states*, where regulatory driving forces vanish.

***F*** contains non-linear terms, as is generally the case for functions describing gene-regulatory interactions, and thus can generate a vast number of stable steady-state configurations ***x*** *. Importantly, this multi-stable dynamics (for a certain class of networks to which GRN belong) can produce gradientlike dynamics [59, 60] in that the driving force that push the state ***x*** (*t*) towards ***x*** * can for most regions of the state space be approximated as ***F*** (***x***) = −∇*V* (***x***), even if the system is non-integrable. Thus, it is permissive to represent the multi-stable dynamics by a *quasi-potential landscape* with ‘elevation’ *V*(***x***) at ***x*** and ‘potential wells’, much as Waddington envisioned with his ‘epigenetic landscape’ in which attractors (valleys) correspond to cell types [58, 60].

An essential corollary is that cells can switch between the discretely distinct stable and *inheritable* phenotypes encoded by attractor states ***x*** without change in the genotype. Two non mutually-exclusive modes for cell transitioning from one attractor to another, $x_{a}^{*}\to x_{b}^{*}$ can be considered:

- Due to gene expression noise, state ***x*** fluctuates in high-dimensional space almost randomly around attractor state ***x*** * and can occasionally overcome the attracting force, resulting in cells ‘jumping’ out of the basin of attraction of $x_{a}^{*}$ (first exit) into that of neighboring attractor state $x_{b}^{*}$ , manifest as a stochastic phenotype conversion. Macroscopically, this event appears like that resulting from a random mutation, but differs from it because the new phenotype is latently present as a developmental potentiality (unused attractor) and thus, there is a much higher probability for a single ‘chance event’ to produce a complex self-stabilizing and selectable phenotype [61].
- Because ***F*** (***x***) captures regulation of gene expression, it provides an entry for environmental influences as the ‘regulator’ of ***x***, e.g. via transcription factors responsive to external signals which change the values of parameters in ***F*** (***x***). This modulation alters the topography of the landscape in ways constrained by the form of *V*(*x*), such as lowering the height of an ‘energy barrier’ Δ*V*(*x*) between attractors. Environmental signals thus act as bifurcation parameters that can ‘catalyse’ attractor (phenotype) transitions [55, 62].

Of importance for cancer progression after treatment is that a neighbouring attractor into which a perturbation shifts the cells often encode stem cell-like states that may have evolved for injury response [55].

### 6.2. Current and future challenges

A series of observations made by new technologies, such as ultra-deep tumor sequencing, single-cell transcriptomics and clonal analysis, has recently exposed cracks in the fundament of the Darwinian somatic mutation theory of cancer [36, 56, 57]. Single-cell transcriptomics data is commonly displayed such that each cell is a dot in some dimension-reduced space (typically, a 2D plane) at position ***x*** of its state ***x***. These points form ‘clusters’ that represent cells in the same attractor, with the dispersion reflecting gene expression noise. Treatment stress imparts a broad perturbation to the GRN of each individual cell, affecting their state ***x*** differently. This increase of cell population dispersion is manifest as broadening of the cell clusters or the increase in the number of clusters (=occupied attractors). Thus, treatment can push cells into nearby attractors some of which may encode their developmentally neighbouring stem-like phenotypes [36, 53, 63, 64].

With single-cell resolution measurement we can observe the temporal change of *N* individual cells in state ***x***, i.e. the ‘cell number density’ *N*(***x***, *t*) and can write for the temporal evolution of the distribution of the cell states ***x*** a Fokker–Planck type equation:

(1) $$\frac{\partial N(x,t)}{\partial t}=\nabla \cdot [D(x)\nabla N(x,t)-F(x)N(x,t)]+g(x)N(x,t).$$

This equation considers the probability of cells, due to stochastic gene expression, to be in state ***x*** (diffusion term in equation (1) with diffusion *D* (***x***)) and the cell state change driven by multi-stable GRN dynamics(second=drift term ***F*** (***x***)); a third term not encountered in systems with mass conservation, captures changes of the number of cells in state ***x*** with growth rate constant *g* (***x***).

*The Darwinian and Lamarckian dynamics are both contained* in equation (1): if the dynamics of *N* (***x***, *t*) is driven mostly by the growth rate *g* (***x***) that differs between for various phenotypes ***x***, we have Darwinian selection. If change is mostly due to the drift term ***F*** (***x***) that can be modulated by environmental regulation, we have Lamarckian induction. Thus, the Darwinian and Lamarckian schemes represent extremes of the same underlying behavior [63, 65]. The challenge is to predict and measure *N* (***x***, *t*).

### 6.3. Advances in science, technology and mathematics to meet challenges

Obviously, it is not realistic to expect to soon know the specific form of ***F*** (***x***). But we can still make sense of burgeoning single-cell transcriptome (or proteome) data *X* (*T*) with

(2) $$X(T)=\left[\begin{array}{ccc} x_{1}^{1} & \cdots & x_{1}^{c}\\ \vdots & \ddots & \vdots \\ x_{m}^{1} & \cdots & x_{m}^{c} \end{array}\right]$$

by analyzing it through the lens of GRN and cell population dynamics without invoking ***F*** (***x***). Here, $x_{i}^{j}$ is the transcription level of gene *i* in cell *j* for a total of *m* genes and *c* cells. The data matrix *X* (*T*) is a snapshot of the states of cells in a population, measured in condition *T* where *T* can be time points in tumor ‘evolution’, e.g. before and after treatment. We consider *X* (*T*) to represent the *c* cells within one (unimodal) cluster (in one basin of attraction). Its population structure is manifest in the distribution of the cell vectors *u* ^1^, *u* ^2^, …, *u* ^*c*^ (columns in *X* (*T*)). But of importance are also the gene vectors *v*_1_, *v*_2_, …, *v*_*m*_ (rows in *X* (*T*)) which reflect the GRN dynamics, as explained below.

The data structure of *X* (*T*) must somehow manifest the underlying dynamics of *F* (***x***). The property that near ***x*** * the cells descend to an attractor state ***x*** * in a (nearly) gradient-driven fashion, allows us to formulate, without *F* (***x***) but using permissive approximations and assumptions (linearization, discretization in time, ergodicity, hyperbolic attractor) and the reversion of a perturbed state ***x*** to ***x*** * (where ***x*** = ***x*** * + Δ ***x***), the dynamics in terms of the Jacobian *J* of *F* (***x***) at ***x*** * [62]:

(3) $$\Delta x(t+1)=\Delta x(t)J\left(x^{*}\right).$$

We are interested in destabilization of the attractor because treatment involves the destabilization of the attractor that cancer cells inhabit, causing cells to exit it and enter the basin of attraction of an apoptosis state [55]. However, attractor destabilization in a multi-attractor landscape also means loss of control, such that some cells may aberrantly ‘spill’ into nearby attractors encoding stem-cell like phenotypes that become accessible [55]. This would explain the inevitable adoption of stem-like phenotypes in cells that survive harsh treatment.

With (additive) gene expression noise and above approximations and assuming *X* (*T*) to represent a snapshot sample of cells fluctuating around ***x*** *, we link the *statistics* to the *dynamics*: *E* (***x***) = ***x*** * that is, the attractor state is (approximately) the expected value *E* for ***x***. Connecting *X* (*T*) to the dynamics expressed as *J*(***x*** *) in equation (3) via its eigenvalues *λ*_*i*_ and using *E* (***x***) = ***x*** * we ask: what happens to cell vectors *u* ^*j*^ and gene vectors *v*_*i*_ in *X* (*T*) during destabilization when the largest eigenvalue λ_*_ goes from λ_*_ < 0 to zero (or 1 for discrete models)?

It is intuitive and can be shown that destabilization of the attractor increases the dispersion of cells, as experimentally confirmed [62]. Thus, the correlation between cell vectors *u* ^*j*^ on average decreases, making the cell population more heterogeneous.

For gene vectors *v*_*i*_ in *X* (*T*) the interpretation is less intuitive. One can show that for λ_*_ → 0 the average correlation between pairs of gene vectors *v*_*i*_ increases towards a maximum at the bifurcation point [62, 66]. Thus, the gene vectors align as the attractor states destabilize in the direction of the eigenvector of *J* (***x*** *) corresponding to λ_*_.

In summary, generic dynamical systems principles without a specific model suggest that as destabilization of an attractor towards a bifurcation proceeds, dispersion of cell vectors and alignment of gene vectors in the data set *X* (*T*) increase. These two changes are manifest as decrease or increase of the average Pearson correlation 〈|*R*(…, …)|〉between the cell vectors *v* ^*j*^ or the gene vectors *u*_*i*_, respectively. We can summarize this as a ratio *I*_*C*_(*T*) that increases when the attractor state destabilizes towards a bifurcation [62]:

(4) $$I_{C}(T)=\frac{\langle \left|R\left(u_{i},u_{j}\right)\right|\rangle }{\langle \left|R\left(v_{i},v_{j}\right)\right|\rangle }.$$

An increase in *I*_*C*_(*T*) or just in gene-gene correlation has been observed in various single-cell transcriptome experiments of cell state transitions [62,66,67]. *I*_*C*_(*T*) may thus be used to identify tumors in the process of destabilizing and acquiring a new phenotype, such as stemness.

### 6.4. Concluding remarks

The single most daunting challenge in treatment of invasive cancer is the near-inevitable recurrence of a more resilient cancer. The need to embrace gene regulatory dynamics, manifest in non-genetic plasticity of cell phenotype, to complement Darwinian somatic evolution of the cancer cell is increasingly appreciated. But single-cell resolution molecular profiles of tumors are still overwhelmingly analyzed using ad hoc, descriptive, heuristic computational algorithms detached from theory or biological first principles. While scientific ‘bottom up’ models of the GRN to predict patterns in the data remains unrealistic, we can solve this dilemma by engaging in *coarse-grained approaches*, still grounded in principles of how biological systems work to identify meaningful structures in the data.

Cancer progression is more than ‘survival of the fittest (cell)’ and its study in terms of fundamental principles of dynamical systems, even without the specific details, may help in designing therapeutic control of a complex non-linear behaviour that too often generates cells in stem-like states upon cytotoxic perturbation.

### Acknowledgments

SH wishes to thank many colleagues for helpful discussions, notably Joseph Zhou and Hong Qian. Unfortunately, due to space limitations only a highly selected number of references, mostly review papers, could be cited—much less then would have been due. This work was funded by the National Institutes of Health through the Grants R01GM109964 and R01GM135396.

## 7. Towards multi-scale mechanistic models of phenotypic plasticity in metastasis and drug resistance

HariKishoreJollyMohit KumarCentre for BioSystems Science and Engineering, Indian Institute of Science, Bangalore, India

### 7.1. Status

The concept of clonal genetic mutations has largely dominated cancer biology research. However, in the past two decades, a focus on phenotypic plasticity as a driver of cancer has emerged thanks to the increased understanding of two critical aspects of cancer: metastasis and drug persistence.

Metastasis remains the cause of more than 90% of cancer-related deaths. Despite the extensive efforts, no unique genetic changes (mutations) have been associated with metastasis. Instead, cellular/phenotypic plasticity—the ability of metastasizing cells to adapt to the repertoire of dynamic adverse conditions that they face and doing so in a fast and reversible manner—has been emerging as a hallmark of metastasis [68]. Cellular plasticity in metastasis takes various forms. The most well-studied among them is epithelial–mesenchymal plasticity (EMP) [69], a developmental process that involves cells dynamically acquiring a spectrum of phenotypes ranging from an adherent, low-motility phenotype (epithelial) to a less-adherent, more-motile (mesenchymal) one. Other ‘axes’ of plasticity that are intricately coupled to EMP include stemness and metabolic reprogramming [70].

In drug evasion scenarios, phenotypic plasticity manifests as drug-tolerant persisters (DTPs). As a phenomenon, persistence is extensively observed and studied in bacterial systems. When bacteria encounter stressful conditions, such as antibiotics, they undergo a phenotypic transition involving a decelerated cell cycle and metabolism while not altering their genetic makeup [71]. This, of course, is not the only mechanism of survival, others being resistance (acquisition of mutations that provide selective advantage) and tolerance, but persistence has the least response time of all. An interesting aspect to note here is that every bacteria cell in a population can achieve persistence. However, only a fraction of cells achieves it in a given time. Furthermore, isolating and re-populating persister cells and exposing them to adversity leads to a similar fraction of persisters in the population as before [71].

Similarly, in cancer, the presence of therapy-evading cancer cells has been noticed for over three decades. However, the classification of these escapees into resistant and persistent cells has only been made possible recently by technological advancements. While separating tolerance from persistence is hard in cancer, reversible tolerance to treatment has been noted through cancer DTPs which do not involve changes in cell’s genetic makeup [72]. Depending on the treatment administered, cancer DTPs can have various characteristic functions: decelerated cell cycle, adaptive cell metabolism, transdifferentiation, or hijacking their micro-environment. These DTPs can serve as reservoirs of cells that can often lead to genetic ‘resisters’ that can survive therapy at long timescales, as they can ‘buy time’ to hedge their long-term ‘solutions’ [50].

Mechanisms regulating cellular plasticity are collectively termed ‘epigenetic.’ They can be broadly divided into two categories: molecular/chromosomal epigenetics (covalent changes at the chromatin structure that control access to the promoter/enhancer regions, thus controlling expression and protein levels) and non-chromosomal epigenetics (stochasticity, cell cycle differences, regulatory networks at transcription, translation, signal transduction levels etc) [73].

In metastasis, multiple experimental and computational studies have identified that complex regulatory networks underlying EMP across cancer types can lead to a spectrum of inter-converting cell states, suggesting non-chromosomal epigenetic regulation [74]. A common theme emerging from preclinical and clinical observations is that the more ‘plastic’ hybrid epithelial–mesenchymal phenotypes are ‘fitter’ for metastasis [75]. Recent data from ChIP-Seq, ATAC-Seq etc has begun to map the chromosomal changes that can work in tandem with non-chromosomal mechanisms during EMP. For instance, presence of ‘master’ EMT-inducing and MET (the reverse of EMT)-inducing epigenetic factors at active chromatin can give rise to a bistable system emerging from concentration variations in these antagonistic factors [76]. Detailed dynamic understanding of such mechanisms is crucial to better decode EMP.

While cancer DTPs can arise from non-chromosomal mechanisms as well, chromosomal epigenetic mechanisms have been extensively reported. Many of these chromosomal changes can be inherited over a few cell generations, thus allowing for the inheritance of persistence and thereby enhanced survival. Epigenetic factors such as SETDB1 in lung cancer, KDM6A in GBM, and KDM5B in melanoma have been associated with persistence. In DTPs, how these factors influence many downstream processes, such as the expression of cell-cycle related genes, is well-studied [50]. However, it is unclear how these factors get recruited precisely at appropriate chromosomal locations to execute corresponding functions.

### 7.2. Current and future challenges

To curb cellular plasticity and consequently cancer aggressiveness, we need to understand the underlying epigenetic mechanisms. The following challenges arise in doing so.

- *Multi-tier regulation in non-chromosomal epigenetics*: multiple non-chromosomal mechanisms have been shown to underly metastasis, including transcriptional, translational, and metabolic regulation etc. Cellular plasticity is an emergent behavior of these regulatory modules. While these modules are being studied individually, a systems-level understanding of these regulatory modules is lacking.
- *Regulatory and inheritable mechanisms of chromosomal epigenetics*: chromosomal or molecular epigenetics involves modifying chromosomes via various mechanisms, including DNA methylation or acetylation. While the downstream effects of these factors have been well documented in EMP and persistence, mechanisms of regulation of these factors are relatively unclear. Given the shorter timescale of epigenetic mechanisms of adaptability, their inheritance ensures maintenance of adaptability for a longer time until the ‘desired’ mutation can be acquired. However, mechanisms and timescales of such inheritable epigenetic ‘memory’ require further decoding.
- *Multi-axial plasticity*: cellular plasticity has multiple interconnected flavours. Hybrid E/M phenotypes have been shown to have higher stemness and drug recalcitrance [75, 77]. Similarly, in EMP, the two types of epigenetic regulations discussed above are often seen to act in tandem. Prolonged exposure to EMT inducer can not only drive the cancer cell population towards a mesenchymal phenotype but also can induce epigenetic locking of phenotypes via chromosomal modification [78]. In the case of drug persistence, epigenetic alterations and intracellular signaling together drive properties such as metabolic adaptivity [50]. Hence, it is crucial to understand these interactions between these different axes of plasticity by integrating mechanistic models with high-throughput data.

### 7.3. Advances in science, technology and mathematics to meet challenges

Mathematical models have made significant contributions in generating new hypotheses and testable predictions to guide experiments. Many such models have been constructed to test different epigenetic mechanisms and their implications in regulating cellular plasticity. Classic models of epigenetic regulation deal with a beads-on-string model of a chromosome, where each bead is a nucleosome and can have one of these states: unmodified, acetylated and methylated. Dodd *et al* proposed the balance between cooperativity and noise in recruitment as a possible mechanism to induce epigenetic ‘memory’, which can then help in the faithful inheritance of epigenetic state of chromosomes across cell generations [73]. In another attempt, Sandholtz *et al* [79], using Hi-C data, have shown that selective binding of HP1 to methylated regions can help in nucleosomes regaining their parental methylation patterns upon replication. How these patterns are affected upon EMP to understand the emergence of phenotypic plasticity across cell generations remains to be investigated. Jia *et al* showed through a mathematical model that recruitment of epigenetic factors upon EMT induction could help fix the state in absence of the inducer, especially upon a prolonged exposure to the inducer [78]. Thus, decoding the dynamics of cellular plasticity across scales of length, time, and regulation is essential to decoding hallmarks of metastasis and drug resistance.

Collection and analysis of high-throughput data at bulk and single-cell level (RNA-seq, ChIP-seq, ATAC-seq, etc) is feasible now; thanks to our advanced technological and computational prowess. Efforts are being made to collect and integrate data at multi-tier regulation levels [80].These advancements, together with the mathematical models, can help in decoding both the underlying design principles and perturbation strategies for the interconnected multi-scale regulatory interactions underlying cellular plasticity [81].

### 7.4. Concluding remarks

**Figure 7.**
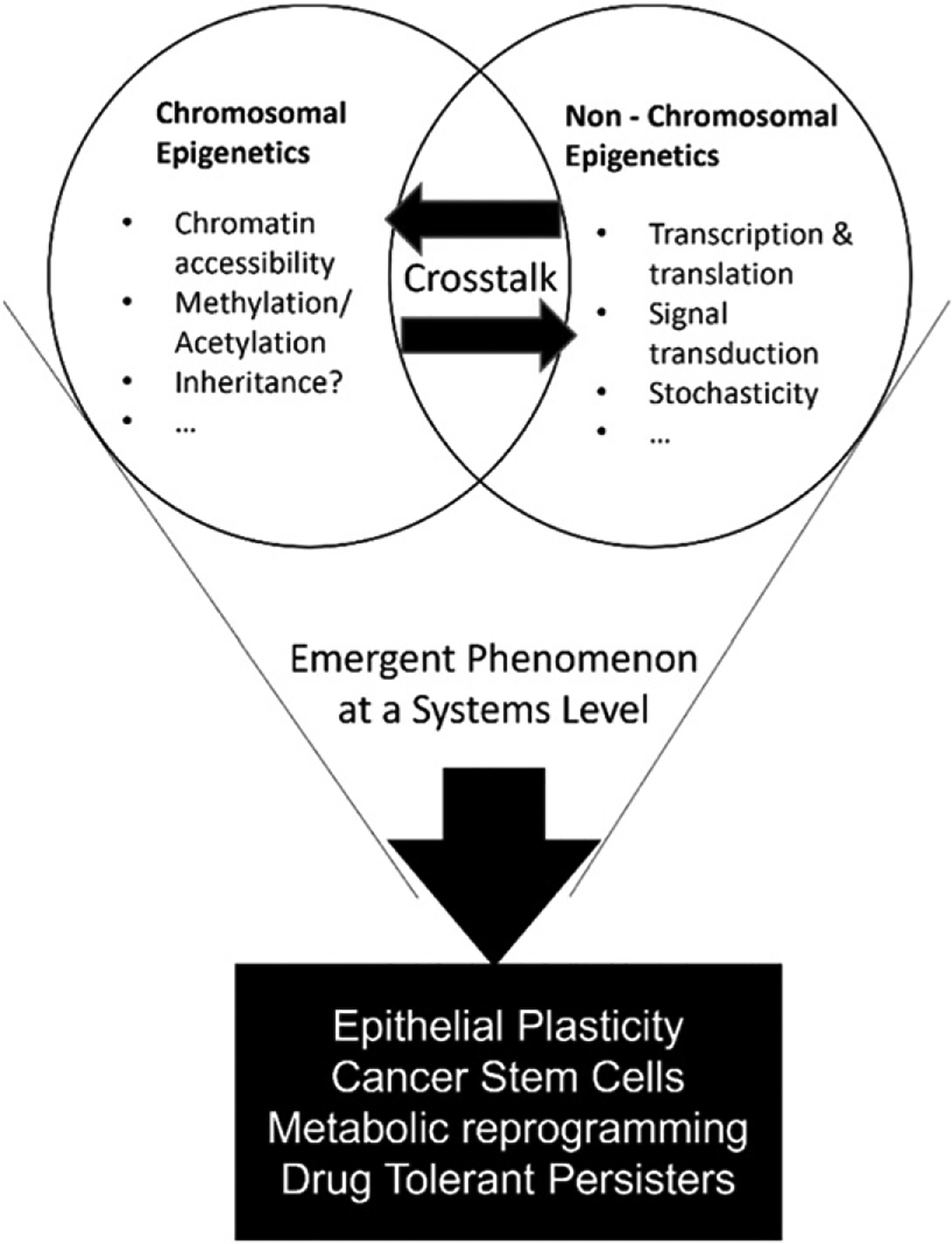
Crosstalk among the chromosomal and non-chromosomal epigenetic arms can drive emergent phenomenon in cancer cells, enabling phenotypic plasticity in many interconnected dimensions/axes.

Integrative approaches involving mechanistic models and machine learning are now being developed to identify patterns in the plethora of data available. This integration can provide a platform to establish causal connections among multi-tiered and multi-modal dynamic data and characterize the epigenetic (figure 7) (both chromosomal and non-chromosomal) regulation dynamics in cancer, with valuable contributions towards designing new rational therapeutic strategies.

### Acknowledgments

MKJ was supported by Ramanujan Fellowship awarded by Science and Engineering Research Board (SERB), Dept. of Science and Technology (DST), Govt. of India (SB/S2/RJN-049/2018). KH was supported by Prime Ministers’ Research Fellowship (PMRF), Govt. of India.

## 8. Operating in the unknown: enabling clinical predictions when we partially understand phenotypic plasticity regulation

HatzikirouHaralamposMathematics Department, Khalifa University, PO Box 127788, Abu Dhabi, United Arab EmiratesCentre for Information Services and High-Performance Computing, TU Dresden, 01062, Dresden, Germany

### 8.1. Status

Phenotypic plasticity has been recognized as one of the main factors that contributes to tumour heterogeneity and eventually in cancer progression and therapy resistance [82]. Many phenotypic plasticity mechanisms have been identified such as the Warburg effect, the epithelial–mesenchymal transition (EMT/MET), migration/proliferation plasticity (Go or Grow) etc. For example, the latter implies that the propensity of motile phenotypes is reduced at the expense of proliferative ones and vice-versa.

Mathematical modelling has been proven instrumental in understanding the impact of phenotypic plasticity mechanisms in tumour progression, growth dynamics or designing appropriate therapeutic approaches. In the case of migration/proliferation plasticity we have shown the existence of an emergent Allee effect for low grade glioma tumours [83] and no ‘one size fits all’ therapeutic approach can be implemented in high grade gliomas [84]. However, developing such models involves a number of reasonable assumptions since not all molecular regulation pathways of the different phenotypic plasticity mechanisms are known. Although modelling insights enhance our qualitative understanding of how phenotypic plasticity impacts disease dynamics, their translation to reliable clinical predictions faces important challenges.

### 8.2. Current and future challenges

In clinical reality, the need of quantitative tumour growth and progression predictions is pivotal for designing individualized therapies. To achieve this a plethora of examinations is conducted to assess the tumour lesion state, spanning from blood sample analysis, clinical imaging (e.g., CT, MRI), biopsy sampling, -omics screening etc. Such medical data correspond to snapshots in time of the patient’s state and in the current standard of care their collection relies on patient’s clinical presentation. This implies that we cannot acquire many data timepoints hampering the personalized calibration of mathematical models and their corresponding prediction potential. Moreover, many clinical data types are not useful in informing phenotypic plasticity models hindering their clinical applicability.

In a nutshell, the use of phenotypic plasticity models in the current cancer standard of care faces the following challenges: (C1) data collection is sparse in time since it relies on patient’s clinical presentation, (C2) we lack the knowledge of the precise pathways involved in regulating phenotypic plasticity mechanisms, and (C3) medical data cannot always inform mathematical models. Overcoming the afore-mentioned challenges to predict the future of a disease and propose an appropriate treatment (e.g., choice of a drug targeting proteins expressed in the tumour) is a formidable but not impossible task.

### 8.3. Advances in science, technology and mathematics to meet challenges

In this section, I present two different approaches that can address the above challenges.

#### 8.3.1. A top-down approach

**Figure 8.**
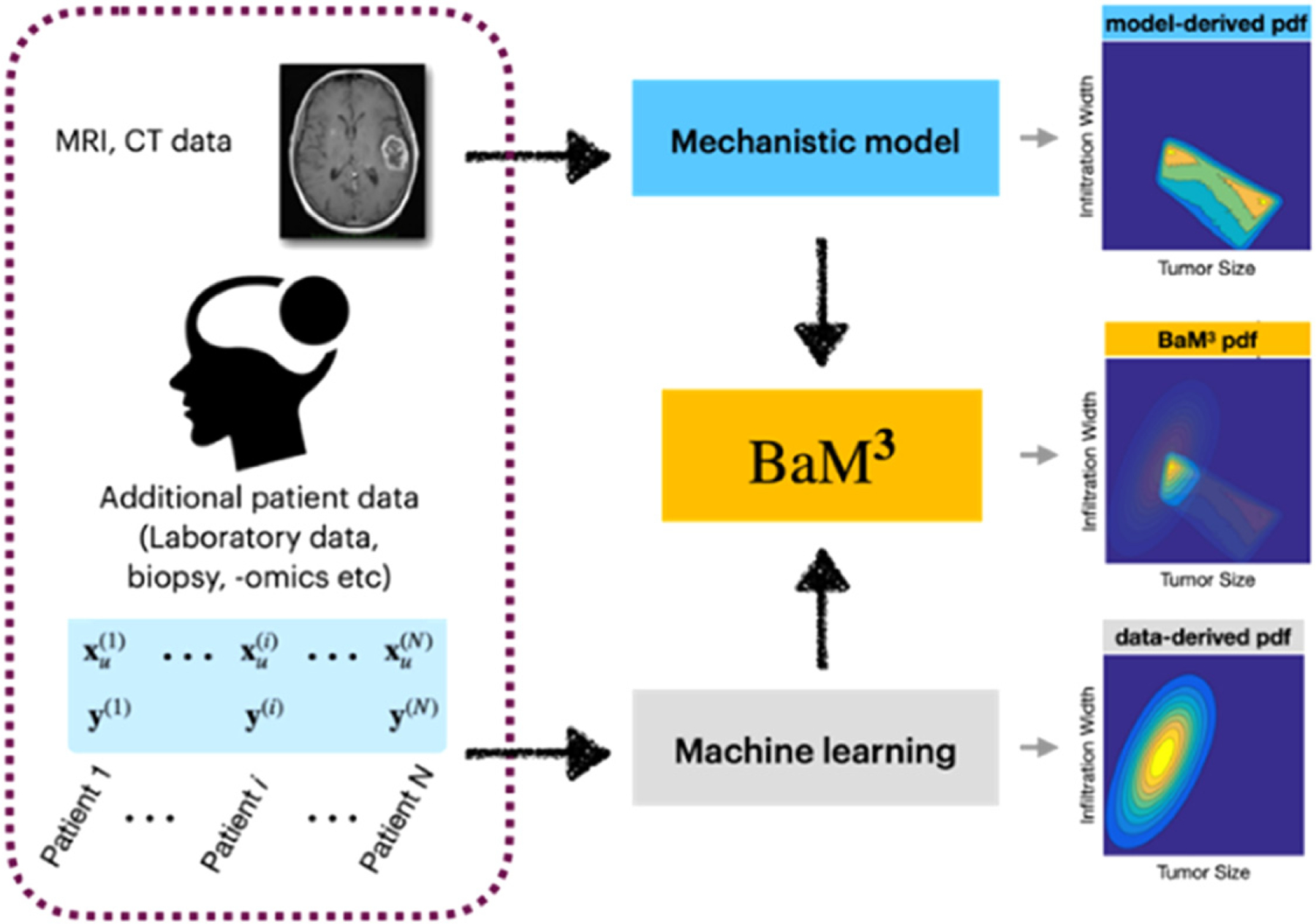
The BaM^3^ method. A schematic representation of the data and method integration of the BaM^3^ method. Details can be found in [86].

The first approach involves the development of methodologies that combine dynamic modelling and machine learning allowing for heterogeneous data integration and enabling predictions under partial biological/mechanistic knowledge. The so-called physics (here biology)-informed machine learning holds the promise of revolutionizing the field of engineering and quantitative sciences [85]. In particular regarding clinical tumour predictions, we have developed a Bayesian combination of machine learning and mechanistic modelling (BaM^3^) [86] that allows for improved clinically relevant predictions (see figure 8). The method uses mechanistic model predictions as intelligent priors, even when mechanisms and parameters are partially known (C2). In turn, it corrects model predictions by harnessing the predictive power of infrequent non-modellable data (C1, C3). We demonstrated BAM^3^ potential on a synthetic dataset for glioma and two real cohorts of patients with leukaemia and ovarian cancer. Predictions from the method are in close agreement with actual clinical data for individual patients, suggesting its potential applicability in enabling accurate personalised clinical predictions. The only limitation of the BaM^3^ framework is related to the fact that the probability distribution of unmodellable data should be in a quasi-time invariant, otherwise prediction quality is hampered (for more details see [86]).

#### 8.3.2. A bottom-up approach

An alternative and ambitious approach to address (C2), i.e., when regulatory mechanisms of phenotypic plasticity are not fully known, is to focus on potential principles that dictate cell decision-making. Such principles have been proposed by the pioneering work of W Bialek [87]. The starting point is how single cells process microenvironmental information. Regarding cells as energetically constrained Bayesian decision-makers that infer their phenotype according to microenvironmental cues, such as other cell type densities, ligands, chemical concentrations, ECM densities, expressed proteins, spatial transciptomic data to name a few, we have recently proposed *least environmental uncertainty principle* (LEUP) [88, 89]. According to LEUP cell phenotypes change to minimize the entropy, i.e., uncertainty, of their corresponding microenvironment. Microenvironmental entropy can be regarded as a potential functional in the sense of Waddington’s epigenetic landscape.

LEUP can be used for developing agent-based models (bottom-up approach) of tumor development, where single cells stochastically decide over their phenotype according to LEUP. This will allow for integrating the existing cell plasticity regulation mechanisms and fill the knowledge gap by the implementation of LEUP. Such LEUP-driven models may produce reliable simulations able to shed light in the role of phenotypic plasticity in tumor progression dynamics and in the design of new therapies.

Currently, LEUP has been used to explain collective migration patterns of spherical *Serratia marcescens* bacteria [90] and the robustness of avian photoreceptor mosaic patterns [91]. In both applications, the common denominator was the partial knowledge of the involved mechanisms regarding bacteria migration direction decisions and photoreceptor fate selection.

**Figure 9.**
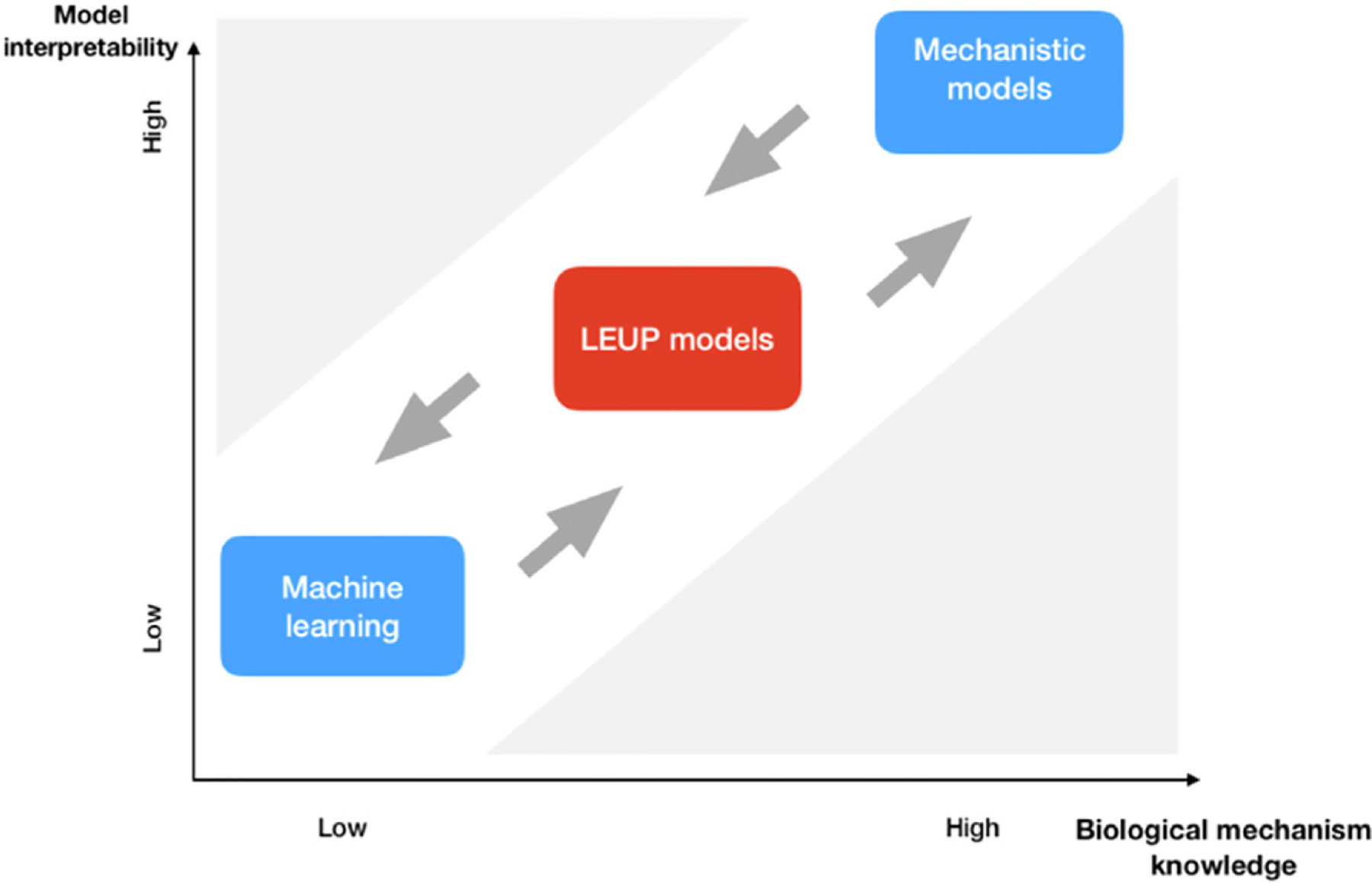
LEUP features. LEUP allows for predictions even when lacking exact mechanistic knowledge. Machine learning offer solutions in similar situations. However, LEUP models are still more interpretable and facilitate generalisation.

Predicting cell phenotypic dynamics using LEUP works as any other entropy maximization method by integrating raw data and prior mechanistic knowledge in the form of optimization constraints. Interestingly, LEUP inferred dynamics offer a good compromise regarding model interpretability and required mechanistic knowledge when compared to machine learning and detailed biophysical models, as shown in figure 9. Although this approach is promising, it still requires significant research in order to be validated and further tested against real data, before becoming useful in a clinical setting.

### 8.4. Concluding remarks

Although phenotypic plasticity mechanisms have a critical impact in tumour heterogeneity and therapy design, their regulation is not always fully known. This fact makes clinical predictions a formidable task. Here, I have presented two approaches to deal with this challenge: (i) the combination of mechanistic modelling of phenotypic plasticity with machine learning and (ii) focus on the principles that dictate cell decision-making and in particular phenotypic plasticity. Currently, the former offers ready to go solutions for clinical implementation, where the latter requires further research.

### Acknowledgments

HH would like to acknowledge the VolkswagenStiftung funding within the Life? program (96732) and he is supported by MiEDGE (01ZX1308D) of the ERACOSYSMED initiative. Moreover, HH acknowledges the support of the FSU Grant 2021–2023 Grant from Khalifa University.

## 9. Can therapeutics keep pace with tumor plasticity? An opportunity for model-assisted learning cycles

PoelsKamrine E^1^SpilkerMary E^2^ShtyllaBlerta^1^
1Early Clinical Development

2Medicine Design, Pfizer Worldwide Research, Development and Medical, United States of America


### 9.1. Status

Tumor plasticity encompasses a vast array of biological mechanisms and its impact on therapeutic response is equally large, leading to resistance against a diverse repertoire of cancer therapies [92, 93]. Elucidation of the primarily factors leading to drug resistance is critical for pharmaceutical decisions regarding clinical drug regimens, combinations, new target selection and drug design requirements. Furthermore, within the pharmaceutical industry, the multi-faceted challenge created by tumor plasticity requires practical and timely action.

While TME, immune involvement, bypass signaling pathways and drug transporters can lead to plasticity and drug resistance, for molecularly targeted agents there has been a significant focus on genetic mutations that render targeted therapies ineffective against cancer cells. As an example, for ALK inhibitors, emerging data following patient treatment with sequential first, second and third generation ALK inhibitors reveal distinct on-target (EML4-ALK) resistance mutation profiles that are dependent upon the therapeutic sequence [94]. A better understanding of genetic mutation evolution is useful to inform optimal therapeutic and drug development decisions. Yet, monitoring the emergence of genetic-driven resistance in treated patients remains a challenge due to heterogeneity in tumors and treatment response. Non-genetic plasticity presents additional complexities that can be particularly difficult to appropriately and efficiently capture in patients. Similarly, at the bench (pre-clinical setting) it can be problematic to elicit, measure or properly define clinically meaningful non-genetic plasticity. Given these emerging complexities associated with tumor plasticity, it is critical that technologies are available to monitor tumor status in patients. In this regard, liquid serial biopsies (i.e., circulating tumor DNA (ctDNA) and circulating tumor cells (CTCs) [95]) are showing promise as a tool to monitor post-therapy genetic and signaling changes in tumors.

Along with robust collection of longitudinal data, we advocate for novel modeling methods that can integrate these serial clinical measurements with other patient data as well as *in vitro* and preclinical knowledge to create a wholistic view of emerging therapeutic resistance patterns to explore alternative therapeutic approaches. Encouragingly, modeling approaches in academia and industry are available to begin this endeavor [96–98].

Here we review some of the challenges and opportunities tumor plasticity presents to oncology drug discovery and development.

### 9.2. Current and future challenges

**Figure 10.**
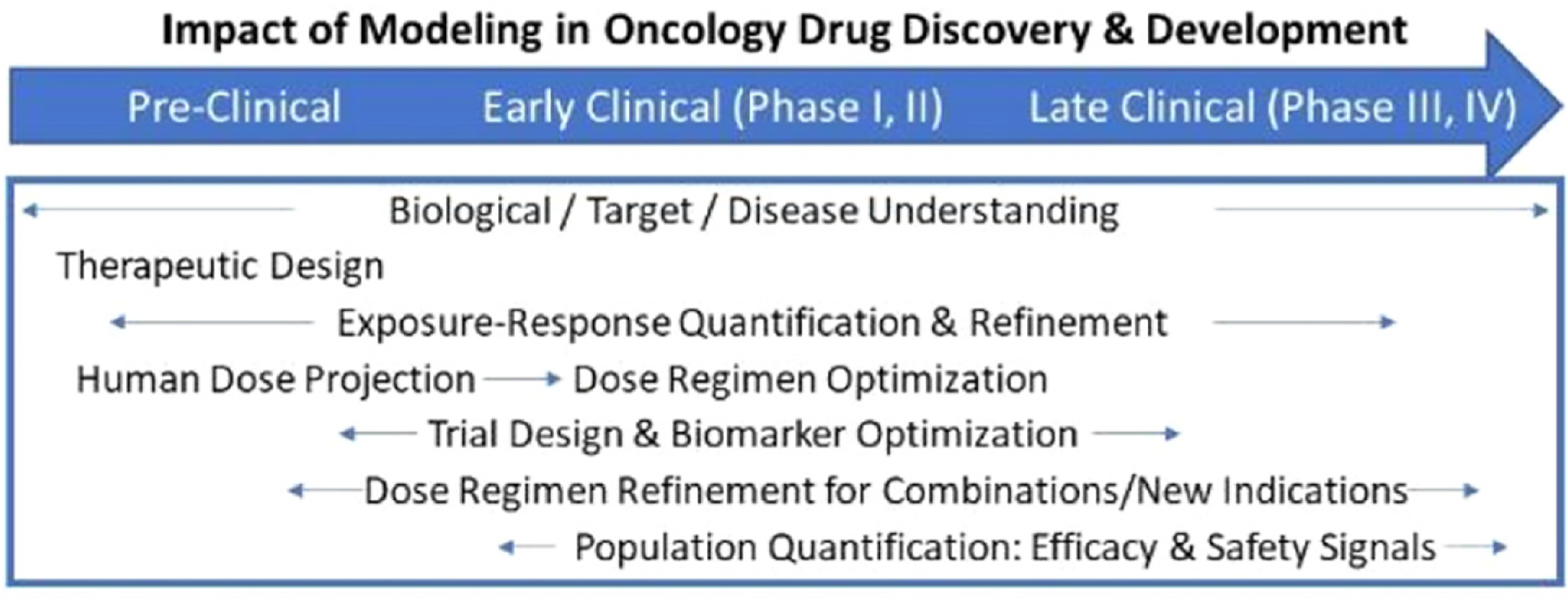
Modeling impact in a pharmaceutical setting.

While modeling approaches are a useful tool to de-risk decisions at various points in the drug-development pipeline (figure 10), especially when dealing with complex problems such as connecting plasticity signals across various datasets, models need to be appropriately calibrated and supported by data.

We outline three main challenges concerning our ability to obtain data that informs our understanding of tumor plasticity and associated drug-tolerant cells [92]. First, most molecular causes that predispose tumor cells to undergo a phenotypic conversion are still unknown, and a stochastic nature of such conversions further complicates our understanding. Second, the sequential dynamics of tumor cell phenotypic plasticity upon treatment are not well understood. Third, the mechanisms influencing plasticity may vary across patients, treatment schedules and disease progression. Thus, ascertaining time-dependent profiles reflective of tumor heterogeneity, plasticity and corresponding drug-tolerant or resistant cells requires advancement and refinement in the resolution of our screening procedures in patients, permitting tumor assessment down to the single cell level [92, 99]. Liquid biopsies appear to be a promising alternative to conventional biopsies, providing both precise molecular data to improve the clinical management of patients (with most notable examples in lung cancer) as well as a less invasive way to sequentially monitor tumor behavior.

Mathematical modeling and early clinical evidence have suggested that repeated detection, profiling and targeting of surviving cells would improve patient outcomes [100, 101]. Liquid biopsies are top contenders for non-invasive and iterative methods to assess resistance/plasticity in the clinic. In this regard, both CTC and ctDNA could assist therapeutic decision-making and supply an adequate reflection of intra-tumor heterogeneity [95].

Iterative collection of ctDNA can address tumor heterogeneity and may predict acquired treatment resistance driven by genetic and epigenetic mechanisms. Methylated ctDNA has been evaluated as a potential liquid biopsy-based biomarker but its application to NSCLC in the clinic is less common than the serial assessment of genetic alterations in ctDNA [102]. Unfortunately, no protein or functional readouts are available from ctDNA data, which could be informative in respect to a tumor’s changing phenotype. Additionally, ctDNA analysis remains limited due to a lack of pre-analytical conditions [95]. In contrast, CTC studies allow for evaluation of cancer phenotype and assist in molecular characterization of the disease. CTCs constitute a small and fragile population of cells with broad heterogeneity, which can make it harder to identify them. However, if successfully captured, they could provide complementary information to that obtained from ctDNA. Unfortunately, both ctDNA and CTC have low signal-to-noise ratio in current screening procedures, especially in early-state disease, so emerging tumor variants may not be detected. This technical hurdle as well as cost and broad accessibility will need to be addressed to improve and better define the clinical utility of these measurements. Importantly, there has been progress in this area in recent years, especially in next-generation sequencing for analyzing ctDNA. The sensitivity of ctDNA detection methods has substantially increased through the optimization of a patient-specific library preparation, and the implementation of novel computational and experimental error correction strategies [96, 103].

### 9.3. Advances in science, technology and mathematics to meet challenges

An emerging challenge is achieving consensus around technical approaches to collect the most robust and reproducible patient data, while also integrating it with insights from pre-clinical *in vitro* or animal data. Mathematical modeling approaches can be useful in bridging these gaps. In the pharmaceutical setting, a range of models are used to guide timely and practical strategies to monitor and optimize tumor response in the presence of treatment, as outlined below and in figure 10.

#### Pre-clinical modeling.

Mathematical modeling in drug discovery informs on basic biological understanding, therapeutic design [104] and ultimately translation of preclinical exposure-response relationships into humans. Emerging clinical data may guide therapeutic opportunities and experiments for model-based quantification of exposure-response relationships through ODE-based PK/PD models [105]. Pre-clinically, tumor complexity is often simplified to permit testing of the therapeutic potential against specific mutations or nodes in cellular pathways; thus tumor plasticity is decoupled into simplified, data-driven, testable pieces.

#### Clinical development: early stages (phase I/II).

Here, models are used to inform selection of the recommended dose for expansion. In addition, virtual clinical trial simulations leveraging quantitative systems pharmacology models can connect clinical biomarkers with pre-clinical biological mechanisms to inform on biomarker selection and study design, while subsequently integrating the collected information for further learnings. **Later stages**. Population PK, PK/PD and disease progression (statistically driven) models are leveraged to define therapeutic performance across a population of individuals.

While early discovery modeling efforts currently focus on identifying and delivering the right compound to the clinic, integrated clinical modeling approaches can impact strategies for minimizing resistance and plasticity when focused on: (i) careful selection of drug regimen and (ii) the use of rational combination treatments that prevent the activation of pathway-compensation mechanisms. We list some examples below that show promise in these two areas.

#### Optimal dose selection.

Historically, the dose finding paradigm in oncology has been dominated by the maximum tolerated dose (MTD) approach wherein phase I dose escalation studies are employed to find MTD using pre-defined dose limiting toxicity criteria. However, this approach has the potential to shift tumors into a stress-response state that encourages resistance of the cells that will survive treatment either due to unequal access to drugs, or heterogeneity of tumor cell phenotypes that encourage escape from treatment. Recent work of Poels *et al* is an example where tumor evolution and resistance modeling are integrated with design of a clinical trial [98]. More work is needed in this space; ecologically inspired adaptive therapies tied to clinical studies from academic groups could have the potential to shift the traditional paradigm and also influence modeling approaches in the pharmaceutical setting [106].

#### Modeling drug combination effects.

Models that incorporate translation of pre-clinical datasets into clinical efficacy projections for multiple drug combinations have incorporated some pathway resistance components [97]. However, translational modeling that can impact tumor plasticity is lacking in this space and we believe this is an area that can be impactful in the near term, particularly as more novel combinations are tested in the clinic.

Finally, we highlight a new generation of models that incorporate novel liquid biomarkers with tumor evolution models [96]. This approach has potential to optimize trial designs, especially if it can be adapted in settings in which monotherapy or combination treatments are included.

### 9.4. Concluding remarks

Robust treatment approaches combating tumor plasticity will require improved monitoring of individual time-dependent patient responses, by promising novel technologies such as ctDNA and CTC. This information in turn can be optimally leveraged in association with mathematical modeling methods. Quantitative models from the academic setting are well equipped to account for new types of data, such as ctDNA, but progress and consensus regarding technical approaches in biomarker data collection and analysis is needed to augment the real impact of these models in the clinic. We hope that a more synergistic union of the intellectual creativity of academicians and the resources from industry will aid in defeating tumor plasticity in the clinic.

### Acknowledgments

K E Poels, M E Spilker and B Shtylla are employees of Pfizer, Inc. These authors have no other relevant affiliations or financial involvement with any organization or entity with a financial interest in or financial conflict with the subject matter or materials discussed in the manuscript apart from those disclosed.

## 10. The importance of phenotypic memory in therapy resistance

Robertson-TessiMarkAndersonAlexander R AIntegrated Mathematical Oncology, Moffitt Cancer Center, Tampa, FL, United States of America

### 10.1. Introduction

Cellular plasticity is one of the driving mechanisms behind the emergence of treatment resistance in cancer. Although the theory of bet hedging has long been studied in many living systems [107–112], it is only in recent years that the idea has been explored in cancer [113–118]. Importantly, a better understanding of stochastic plasticity has the potential to significantly alter the way therapies are delivered. The general principle of bet hedging is that two or more phenotypes are generated within an isogenic population, and these phenotypes have different fitness in different environments. For example, persister cells in bacteria [107, 119, 120] are a phenotype that has low fitness in environments that favor the growth of the primary ‘normal’ phenotype, while having high fitness in toxic environments where the normal bacteria rapidly die. A strain of bacteria may therefore stochastically and rarely produce persister phenotypes, which act as a hedge against a future toxic environment to prevent population extinction.

Here, we focus on phenotypic memory in the setting of bet hedging [117], wherein a population that is using bet hedging can alter its phenotypic probabilities such that recently successful strategies are more favored. Although numerous biological mechanisms could create this memory effect, here we use a chemical reaction network (CRN) to illustrate that rich population dynamics can arise from a very simple memory bet-hedging scheme. This has implications for cancer therapy, especially if mechanisms that foment phenotypic memory can be targeted, which would increase the efficacy of primary agents.

### 10.2. Bet-hedging dynamics

**Figure 11.**
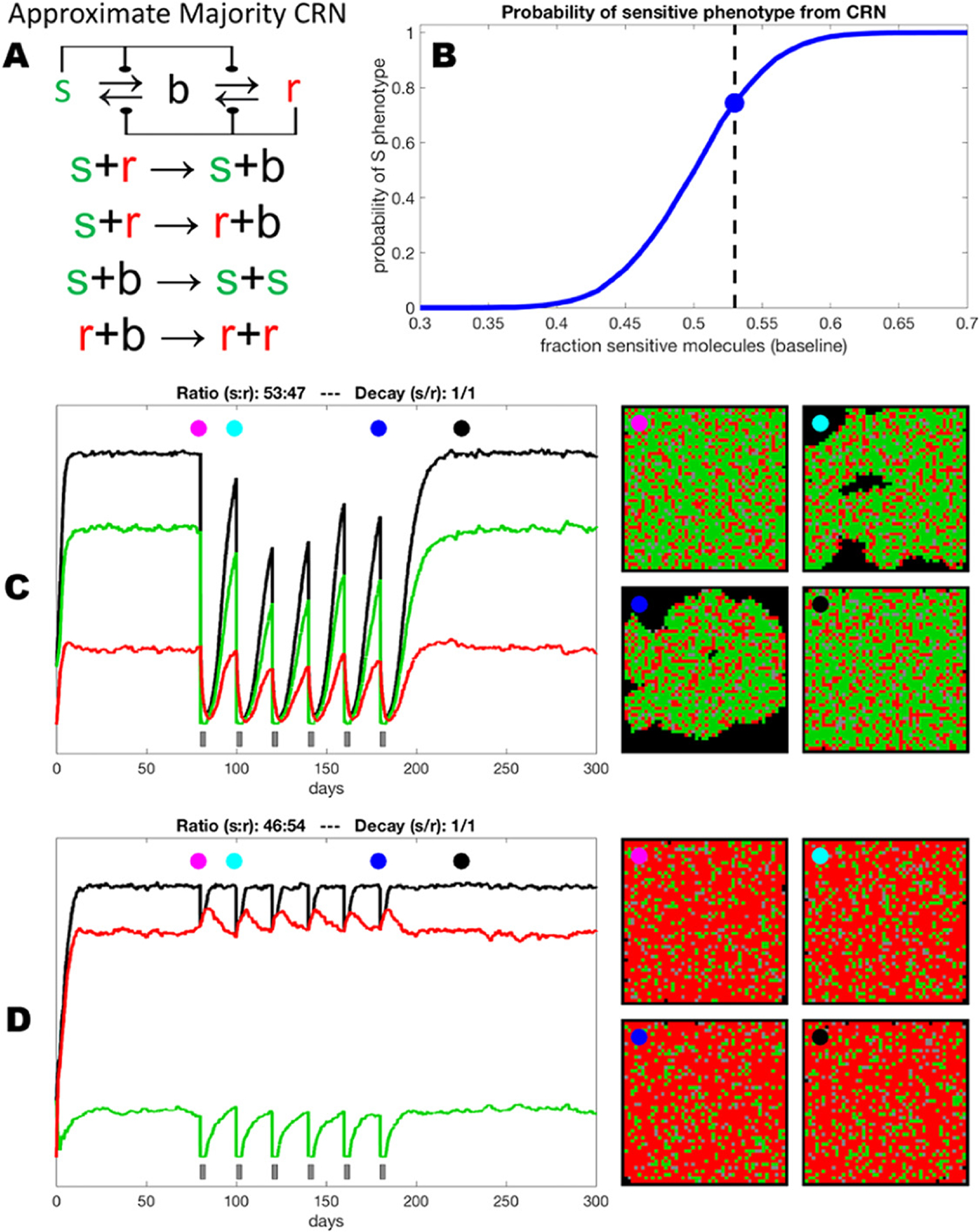
Bet hedging without phenotypic memory. (A) CRN (approximate majority) that is a bistable switch between states of all *s* or all *r* molecules, using a facilitating molecule *b*. (B) Probability that the CRN produces phenotype *S*, for a given fraction of starting *s* molecules. (C) Simulation using 53*s* and 47*r* upon cellular division (dashed line in panel (B)) and treating with six pulses of therapy. (D) Simulation using 46*s* and 54*r*, with the same therapy as panel (C).

We use a parsimonious agent-based model of bet-hedging (without phenotypic memory, to begin with) to illustrate some key behaviors that depend on generalized physical properties of the system (figure 11). The model simulates individual cells that can be in either of two phenotypes: fast-growing 100%-sensitive (*S*, green) or slow-growing 100%-resistant (*R*, red). Upon division, a cell produces two daughter cells, and each can change its phenotype with a probability that is determined by the outcome from the iteration of an ‘approximate majority’ CRN (figure 11(A); see [114] for details). The network is initialized with a certain number of each of two molecules (*s* and *r*, representing the *S* and *R* phenotypes respectively). Note that the *b* molecule is a transient product of the reactions and starts and ends at zero. We define the genotype of a cell as the fixed initial numbers of *s* and *r* molecules produced, and these numbers determine the probability of the daughter cell being phenotype *S* or *R*. For example, if the network commences with 50 of each type of molecule, the CRN will resolve to have 100 molecules of *s* (and therefore an *S* phenotype cell) about half the time, and 100 molecules of *r* (and phenotype *R*) the other half. The CRN is run twice at the time of cell division: once for each daughter cell, to determine their phenotypes independently. Figure 11(B) shows the probability of producing an *S* daughter for different fractions of *s* molecules present at the start of the CRN iterations, given a total of 100 molecules of either type.

We are interested in the dynamics of these populations under therapy (here, six pulses of a drug that kills only *S* cells). Figure 11(C) shows the case with genotype 53*s*/47*r* (which produces about 75% *S* daughter cells). The behavior is like that of a persister population: the *R* subpopulation prevents the species from going extinct. Figure 11(D) uses genotype 46*s*/54*r* (which produces > 80% *R* cells); here, the population is mostly resistant to therapy, representative of multicellular tissue where significant cell death is undesirable.

### 10.3. The impact of phenotypic memory

**Figure 12.**
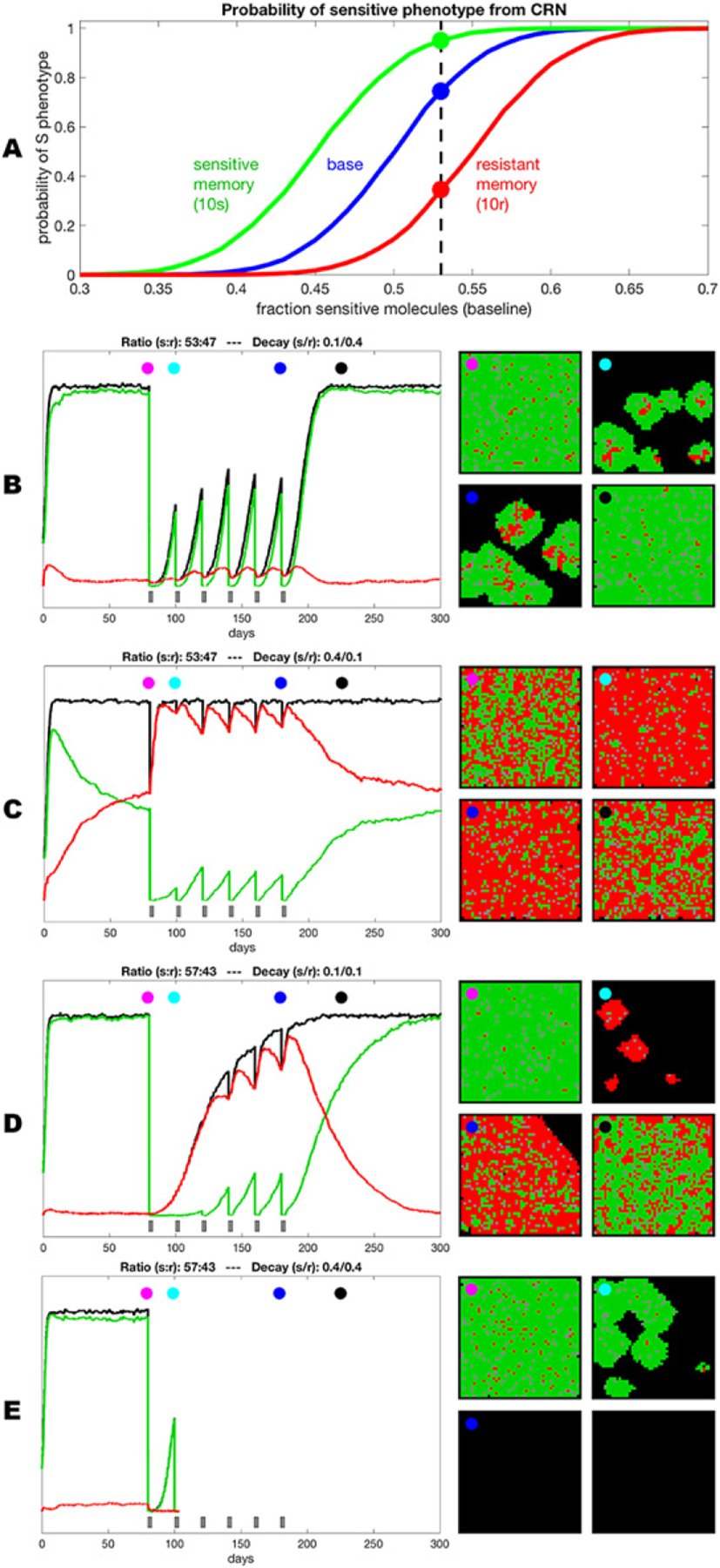
Bet hedging with phenotypic memory. (A) Adding molecular memory and decay shifts the probability curve when the dividing cell still has molecules remaining. Green and red curves show the shifts for 10*s* and 10*r* remaining molecules at division, which are added to the 53*s*/47*r* baseline. This will bias the probability of producing daughter cells toward preserving the parental phenotype. (B) Simulation with 53*s*/47*r*, slow *s*-decay, and fast *r*-decay shows a persister population: there is no sustained relapse during remission, only survival, then rapid regrowth once treatment ends. (C) When decay rates are swapped (*s*-decay is fast and *r*-decay is slow), we see a population that maintains a high density, representative of a multicellular tissue. Unlike in figure 11(D), the off-treatment population has almost 50% sensitive cells. (D) With a different genotype (57*s*/43*r*) and slow decay for both molecules, the population can grow continuously under therapy. Compare with panel (B), where indefinite therapy would hold the population to low-level spikes rather than sustained growth. (E) For some parameters, the population can be driven extinct, suggesting that agents that affect the hedging and decay rates may be powerful combination therapies that could enhance the primary cytotoxic agent’s effect.

A key limitation faced by the above populations is that the fitness of each genotype is not optimal. In the persister-like case (figure 11(C)), the number of *R* cells needed to prevent extinction during therapy is large, and this reduces off-treatment fitness; in the multicellular-like case, the highly fit sensitive cells always have a minor presence, and again the population is poorly fit for growth off-treatment. Ideally, a population would be better served if *successful* phenotypes tended to not switch strategies, while *unsuccessful* phenotypes would favor switching. This is a form of phenotypic memory and can be modeled as follows: rather than reinitializing the CRN with the fixed initial *s* and *r* molecules upon division, daughter cells inherit half the molecules present in the parent at time of division. Importantly, after the CRN is run and phenotype determined, the remaining molecules undergo decay, such that the longer a cell has lived before dividing, the fewer molecules it passes to its daughters. These remaining inherited molecules are then added to the fixed genotype molecules before running the CRN for each daughter; this has the effect of reducing the probability of switching phenotypes from the parent. Figure 12(A) illustrates: the baseline genotype of initial 53*s*/47*r* molecules normally produces about 75% *S* phenotypes. If a sensitive parent has 20 *s* molecules left after some time, the chemical reaction in each daughter will start the CRN with (53 + 10)*s*/47*r* molecules, which will have a much greater probability of producing an *S*-phenotype daughter. Note that the genotype defined by the number of molecules added (53*s*/47*r*) remains the same across cells, and it is the remaining molecules that shift the probabilities from the baseline defined by the genotype alone. Similarly, a resistant cell with 20 *r* molecules remaining at time of division will have a greater probability of producing an *R* daughter than the baseline of 25%. The longer a cell takes to divide (i.e., the lower the proliferative fitness), the fewer molecules remain in the cell and therefore the daughter phenotype probabilities approach the original unbiased probability determined by the genotype. The net result of this system is one that has phenotypic memory, where cells that are dividing more rapidly will tend to keep their phenotype, relative to the baseline chance of switching without memory.

By exploring different ratios of the genotype molecules and their decay rates (figures 12(B)–(E)), a wide variety of phenotypic dynamics can be generated. Key elements of the illustration are that (1) the steady-state ratio of *S* and *R* cells can be fine-tuned through genotype ratio and decay rate; (2) this pre-treatment *S*/*R* ratio affects the initial response due to therapy; and (3) the responses to therapy include a micro-persister strategy (panel (B)), a tissue-preservation strategy (panel (C)), and a hybrid strategy (panel (D)) where the population can grow equally well in both conditions. Figure 12 (E) shows a case where the parameters can even lead to tumor extinction. In the latter, the decay rates are fast, and the probability of producing resistant cells is very low.

An interesting aspect of this system is that evolution can easily act on the properties of these molecules to change their expression levels (e.g., changes to transcriptional control) and their decay rates (e.g., via phosphorylation, localization, mutation, etc). Depending on the desired functionality of the cells and tissue in question, a suitable strategy can be found in the evolutionary landscape that would maximize the fitness of the population subject to treatment (or other perturbations). Importantly, figure 12(E) suggests that agents that alter the mechanisms of phenotypic stochasticity (such as those that target epigenetic controls like HDAC) could be powerful combination therapy agents that improve the efficacy of cytotoxic drugs.

### 10.4. Challenges and opportunities

A key challenge in researching plasticity is that the biology is vastly more complicated than the simple illustration presented above. Cellular networks often use dozens if not hundreds of interacting molecules, which in turn produce many more than two phenotypes; subtle temporal aspects also likely play a significant role, since molecules are constantly being transcribed from the genome and then degraded by cellular processes, complicating the meaning of ‘resolution’ for a CRN. Furthermore, the microenvironment of a cell is also a key input into phenotypic expression. Molecules may be acquired from the environment, either as metabolites or signals produced by other cells, and these can influence the balance of phenotypic outcomes. This effectively acts as an ‘environmental memory’: as cells generate successful phenotypes in a changing environment, they may release signaling molecules that bias nearby cells to switch to the same fitter phenotype with more likelihood, and therefore produce faster population growth than would be seen with independently switching cells.

Along with the advanced experimental techniques needed to study phenotypic heterogeneity, mathematical modeling is a key component to disentangling these complexities. The primary challenge remains in identifying realistic networks, timescales, and mechanistic interactions from the biology.

### 10.5. Concluding remarks

Bet hedging with phenotypic memory can create a wide range of dynamics from stable resistant tissues to small-population persister-type dynamics. These behaviors occur even in a system with only two phenotypes; indeed, it is the way in which these phenotypes arise that leads to the rich variation. Understanding these dynamics will give insight into the process of therapy resistance through plasticity, which in turn can inform epigenetic-based treatments that enhance the effect of existing therapeutic agents.

### Acknowledgments

The authors thank Dan Nichol and Tyler Cassidy for useful discussions. Robertson-Tessi and Anderson gratefully acknowledge funding from both the Cancer Systems Biology Consortium and the Physical Sciences Oncology Network at the National Cancer Institute, through Grants U01CA232382 and U54CA193489 as well as support from the Moffitt Center of Excellence for Evolutionary Therapy.

## Data Availability

No new data were created or analysed in this study.
